# Analysis of CNS autoimmunity in genetically diverse mice reveals unique phenotypes and mechanisms

**DOI:** 10.1172/jci.insight.184138

**Published:** 2024-11-08

**Authors:** Emily A. Nelson, Anna L. Tyler, Taylor Lakusta-Wong, Karolyn G. Lahue, Katherine C. Hankes, Cory Teuscher, Rachel M. Lynch, Martin T. Ferris, J. Matthew Mahoney, Dimitry N. Krementsov

**Affiliations:** 1 Department of Biomedical and Health Sciences, University of Vermont (UVM), Burlington, Vermont, USA.; 2 The Jackson Laboratory, Bar Harbor, Maine, USA.; 3 Department of Neurological Sciences and; 4 Department of Medicine, UVM, Larner College of Medicine, Burlington, Vermont, USA.; 5 Department of Genetics, University of North Carolina at Chapel Hill (UNC), Chapel Hill, North Carolina, USA.

**Keywords:** Autoimmunity, Genetics, Genetic variation, Mouse models, Multiple sclerosis

## Abstract

Multiple sclerosis (MS) is a complex disease with significant heterogeneity in disease course and progression. Genetic studies have identified numerous loci associated with MS risk, but the genetic basis of disease progression remains elusive. To address this, we leveraged the Collaborative Cross (CC), a genetically diverse mouse strain panel, and experimental autoimmune encephalomyelitis (EAE). The 32 CC strains studied captured a wide spectrum of EAE severity, trajectory, and presentation, including severe-progressive, monophasic, relapsing remitting, and axial rotary–EAE (AR-EAE), accompanied by distinct immunopathology. Sex differences in EAE severity were observed in 6 strains. Quantitative trait locus analysis revealed distinct genetic linkage patterns for different EAE phenotypes, including EAE severity and incidence of AR-EAE. Machine learning–based approaches prioritized candidate genes for loci underlying EAE severity (*Abcc4* and *Gpc6*) and AR-EAE (*Yap1* and *Dync2h1*). This work expands the EAE phenotypic repertoire and identifies potentially novel loci controlling unique EAE phenotypes, supporting the hypothesis that heterogeneity in MS disease course is driven by genetic variation.

## Abstract 2

We leveraged genetically diverse mice to expand the phenotypic repertoire of the experimental autoimmune encephalomyelitis model of multiple sclerosis and identify loci controlling disease phenotypes.

## Graphical abstract

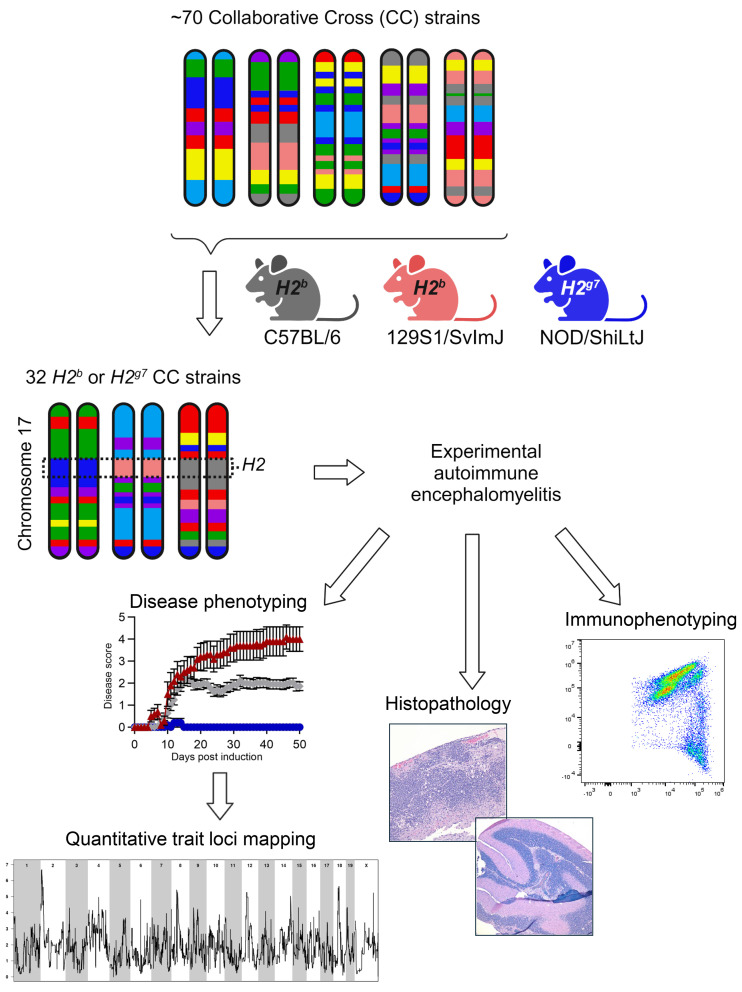

## Introduction

Multiple sclerosis (MS) is an autoimmune disease of the CNS characterized by demyelination, gliosis, axonal loss, and progressive neurological dysfunction, representing the leading cause of nontraumatic neurological disability in young adults (1). Current evidence suggests that activation of myelin-reactive CD4^+^ T cells triggers an inflammatory cascade in the CNS, recruiting other immune cells, which mediate subsequent tissue destruction and pathology (2, 3). Significant heterogeneity in disease presentation and severity exists, with disease courses characterized as: clinically isolated syndrome (CIS) (4), relapsing remitting MS (RR-MS), secondary progressive MS (SP-MS), and primary progressive MS (PP-MS) (5). Additionally, significant sex differences exist; MS incidence is approximately 3 times higher in women, while disease severity is greater in men (6). The biological underpinnings behind this heterogeneity in disease presentation remain largely unknown.

Approximately 30% of MS risk can be attributed to genetic factors (7). Early studies in MS families identified HLA-DRB1*15:01 as the strongest risk allele (8, 9). Subsequent case-control GWAS have identified 200 non-MHC loci and 32 independent loci within the MHC, as associated with MS incidence (7). However, despite the well-characterized genetic association with MS disease incidence/risk, the genetic basis for MS disease course heterogeneity remains poorly understood.

Several animal models of MS exist, with experimental autoimmune encephalomyelitis (EAE) being the principal immune-mediated model. This model has been instrumental in improving our understanding of MS pathogenesis and developing new disease-modifying therapies (10). However, rodent models of MS, particularly EAE, have been criticized for failing to capture many relevant aspects of the human disease (11, 12). We propose that this shortcoming is in part due to the failure to consider the importance of genetic heterogeneity that is so pronounced in human populations — a gap that we attempted to address in this study.

While conventional laboratory inbred strains of mice, such as C57BL/6J (B6), are an important tool in genetics, they represent artificially selected organisms originating from a small founder population (13), lacking the range of genetic diversity and evolutionary pressure in human populations. These limitations can be overcome using so-called wild-derived inbred strains, which are highly divergent from classic strains (14). This approach has been used previously in our lab by leveraging wild-derived PWD/PhJ (PWD) mice and the B6.Chr^PWD^ chromosome substitution strain panel (15). Our studies show that PWD mice have a decreased susceptibility to EAE (16) and demonstrate that PWD-derived alleles profoundly regulate EAE severity, often in a sex-specific manner (17, 18). While B6.Chr^PWD^ mice could be used as a starting point for mapping of specific gene variants driving EAE phenotypes, this is a laborious process. Furthermore, the genetic diversity, although improved, is still limited to 2 allelic variants per gene (PWD and B6) and captured only classic-EAE symptomatology (ascending paralysis). To capture a broader spectrum of disease heterogeneity more representative of MS, we turned to the Collaborative Cross (CC) mouse genetic resource: a panel of multiparental recombinant inbred strains designed for analysis of complex phenotypes (19). These mice were generated using 8 founder strains: 5 conventional laboratory inbred strains — B6, A/J, 129S1/SvImJ (129S1), NOD/ShiLtJ (NOD), NZO/HlLtJ (NZO) — and 3 wild-derived strains — CAST/EiJ (CAST), PWK/PhJ (PWK), and WSB/EiJ (WSB) — collectively covering ~90% of the known genetic variation in mice (19, 20).

Since becoming commercially available, the CC mice have been applied to several research fields, including examination of the genetic susceptibility to infectious diseases and control of immunologic phenotypes (21, 22), but to our knowledge, this model has not yet been applied to study organ-specific autoimmunity. Here, we developed an EAE induction protocol for use in CC mice, using strains carrying *H2^b^* and *H2^g7^* MHC haplotypes. Using this approach, we characterized EAE phenotypes in 32 CC strains. This revealed wide variation in EAE phenotypes and identified several strains with clinically relevant phenotypes, including EAE resistance, severe-progressive disease, RR disease, monophasic disease, and atypical axial rotary–EAE (AR-EAE), as well as the presence of genotype-specific sex differences. Follow-up analysis revealed distinct immunopathology associated with several EAE phenotypes. Quantitative trait loci (QTL) mapping and candidate gene prioritization identified potentially novel loci and genes controlling EAE phenotypes. This characterization expands the phenotypic repertoire of the EAE model, bringing it closer to human disease relevance by addressing the role of genetic diversity in disease presentation. Together with emerging genome-wide studies in humans (23, 24), these findings strongly support the hypothesis that heterogeneity in MS disease course is driven by natural genetic variation.

## Results

### Myelin oligodendrocyte glycoprotein 35–55 peptide–induced (MOG_35–55_–induced) EAE in H2^b^ and H2^g7^ CC strains captures a broad range of clinically relevant disease phenotypes.

EAE, like MS, is initiated by autoreactive CD4^+^ T cells recognizing myelin antigens presented on MHC class II molecules. These autoreactive CD4^+^ T cells are typically elicited by immunization with myelin antigens together with adjuvants such as CFA and pertussis toxin (PTX). Immunogens used for EAE induction include crude mouse spinal cord homogenate (mSCH), recombinant myelin proteins, or, most commonly, peptides derived from myelin proteins, including MOG_35–55_. Different strains of mice have varying susceptibility to EAE immunogens based on the ability of their MHC allelic variants to bind and present different peptides (25). This is problematic to the design of a universal EAE induction protocol for CC strains, as the original 8 CC founder strains contributed 7 different MHC haplotypes (Supplemental Table 1; supplemental material available online with this article; https://doi.org/10.1172/jci.insight.184138DS1). Given this, we initially attempted to induce EAE utilizing mSCH, to capture all potential neuroantigens for binding across various MHC variants. However, experiments in a subset of CC strains demonstrated that mSCH-based induction, although fairly effective in B6 mice, had variable and low penetrance of EAE in CC mice (Supplemental Figure 1).

**Figure 1:**
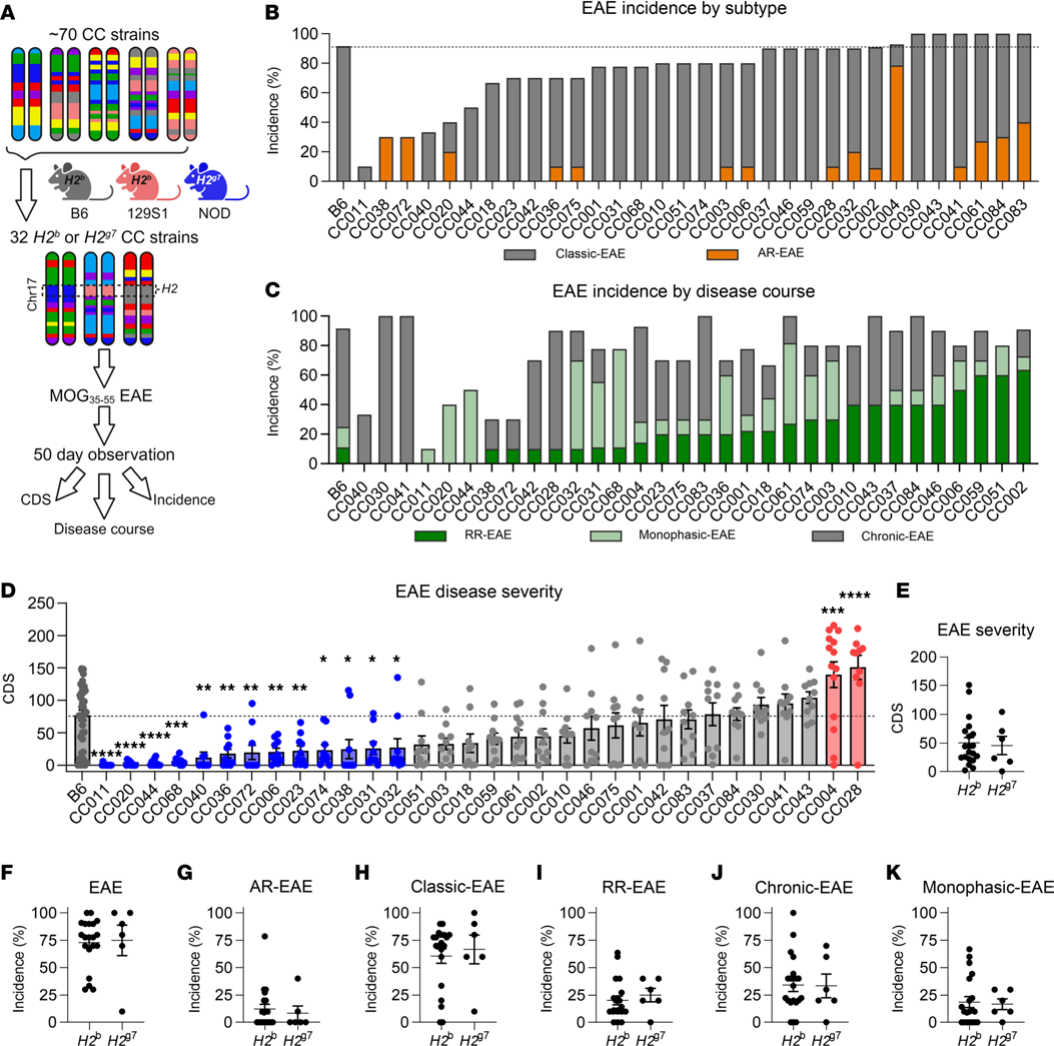
MOG_35–55_ induced EAE in CC strains results in heterogeneous disease profiles. EAE was induced via 200 μg MOG_35–55_ in CFA (s.c.) and 200 ng PTX (i.p.) in 8- to 14-week-old male and female B6 (18 male [M], 18 female [F]) and *H2^b^* or *H2^g7^* CC mice (32 strains, ~5M, ~5F; Table 1). (**A**) Schematic illustrating the study design. (**B**) Percent EAE incidence per CC strain with B6 shown for reference control. Bar color denotes EAE subtype (classic,gay; AR, orange). (**C**) Percent incidence of RR (green), monophasic (light green), and chronic (gray) EAE in CC strains, with B6 shown for reference control. (**D**) Comparison of EAE disease severity in CC strains, as calculated by CDS, versus B6 reference controls. Significance of differences of each CC strain from B6 reference control was determined via 1-way ANOVA with Dunnett’s multiple-comparison test and indicated by asterisks where significant. Corresponding colors indicate directionality as compared with B6 (blue, less severe; red, more severe). (**E**–**K**) Distribution of strain CDS and incidence of EAE, classic-EAE, AR-EAE, chronic-EAE, RR-EAE, and monophasic-EAE, grouped by *H2^b^* and *H2^g7^* homozygous haplotypes. Each data point in **E**–**K** represents a strain average. Significance of differences between haplotypes was determined by 2-tailed unpaired *t* test.

As an alternative, we chose a peptide-based EAE induction approach utilizing MOG_35–55_, based on the identification of compatible allelic variants at the MHC locus (called *H2* in the mouse). Previous studies demonstrated successful MOG_35–55_–based EAE induction in 3 CC founders: B6, 129S1, and NOD (25, 26). These strains carry either an *H2^b^* (B6 and 129S1) or *H2^g7^* (NOD) haplotype (Supplemental Table 1). Using the available CC genotype data, we identified 32 CC strains that carry either an *H2^b^* or *H2^g7^* haplotype (Table 1), which were therefore selected for this study (Figure 1A).

Male and female mice (~5/sex), 9–14 weeks old, of each of the selected 32 CC strains, as well as B6 reference controls, were immunized s.c. with 200 μg MOG_35–55_ emulsified in CFA and 200 ng PTX i.p. as an ancillary adjuvant. Mice were observed daily for a total of 50 days for the presence of clinical disease symptoms using both the classic-EAE (17) and a modified AR-EAE (27) scoring scale as previously described. Daily disease scores (Supplemental File 1 and Supplemental Figures 2–7) were utilized to calculate EAE quantitative trait variables (QTVs) and derive EAE phenotypes (Table 1 and Supplemental Table 2).

**Figure 2:**
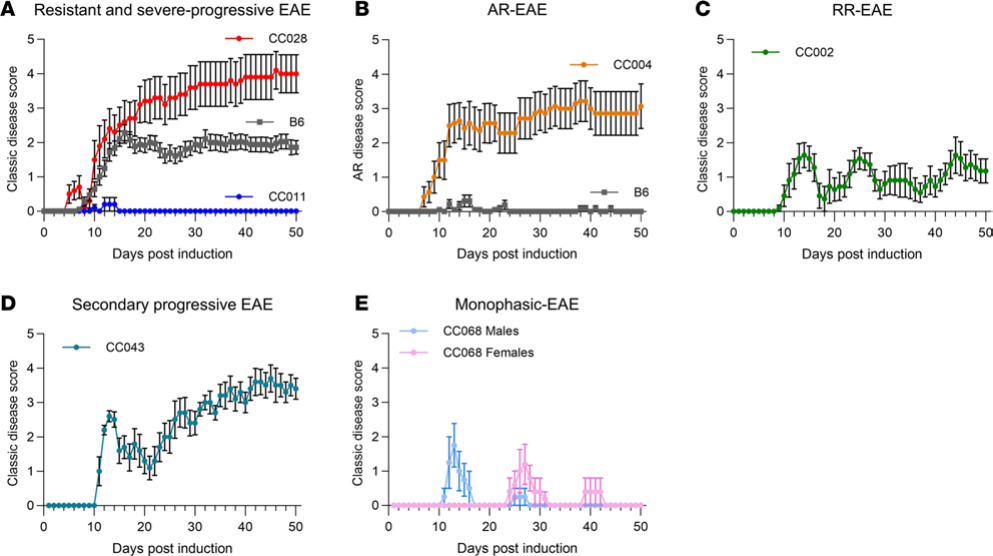
EAE in CC strains captures clinically relevant disease courses. EAE was induced and evaluated in CC and B6 reference control mice as described in Figure 1. (**A**–**E**) Daily strain disease course profiles for strains of interest are shown, including severe-progressive EAE in CC028 (5M, 5F) (red) and EAE resistance in CC011 (5M, 5F) (blue), compared with B6 (18M, 18F) reference controls (gray) (sexes pooled) (**A**); AR-EAE in CC004 (7M, 7F) (orange) (sexes pooled) (**B**); RR-EAE in CC002 (6M, 5F) (sexes pooled) (**C**); secondary progressive EAE in CC043 (5M, 5F) (sexes pooled) (**D**); and monophasic-EAE in CC068 (4M, 5F) (sexes shown separately due to timing of disease onset) (**E**). All panels show classic-EAE scores, except **B**, which shows AR-EAE scores, as indicated on the *y* axes.

In control B6 mice, this protocol resulted in a high penetrance (~92%) of typical symptomatology manifesting as ascending paralysis (i.e., classic-EAE), with a moderately severe chronic disease phenotype (Figure 1, B–D, and Figure 2, A and B). In contrast, CC strains demonstrated a broad range of EAE phenotypes, with the overall incidence of EAE (of any type) ranging from 10% to 100% (Figure 1B, Table 1, and Supplemental Table 2). Analysis of disease course and EAE phenotypic traits classified CC strains into unique disease profiles/subtypes (Table 1). Besides classic-EAE, a number of strains exhibited high incidence of atypical AR-EAE, manifesting as severe ataxia and axial rotation (Figure 1B, Table 1, and Supplemental Table 2). Additionally, a number of strains exhibited RR and monophasic disease (Figure 1C, Table 1, and Supplemental Table 2). We used cumulative disease score (CDS; accounting for both classic and AR-EAE subtypes) as a single quantitative variable capturing overall EAE severity/duration. CDS ranged greatly across CC strains, with several strains demonstrating significantly lower CDS compared with B6 and with 2 strains demonstrating significantly higher CDS (Figure 1D, Table 1, and Supplemental Table 2).

Because MHC class II alleles are the major genetic determinant of susceptibility to MS (8, 9, 28), we asked whether the limited *H2* haplotypes captured influenced EAE. Stratifying CC strains by *H2* haplotype (*H2^b^* or *H2^g7^*; Figure 1, E–K) or by founder strain (B6, 129S1, or NOD; Supplemental Figure 8) demonstrated no significant difference in EAE CDS or incidence of EAE subtypes.

While many CC strains manifested clinical EAE presentation similar to B6, several strains captured extreme ends of the different phenotypes studied (Figure 2, A–D; Table 1; and Supplemental Table 2). These included diversity in both susceptibility and severity, from nearly completely resistant (CC011) to highly susceptible (CC028) — the latter presenting with rapidly progressing severe disease, with 70% (5 of 5 males and 2 of 5 females) reaching quadriplegia/humane endpoint by D39 (Figure 2A). CC004 mice presented with the highest incidence (79%; Figure 1B and Supplemental Table 2) of AR-EAE with a severe chronic disease course (Figure 2B). Additionally, several strains exhibited diversity in disease course. Four strains exhibited a ≥ 50% incidence of RR-EAE, with CC002 being the most robust (Figure 1C and Figure 2C). CC043 mice demonstrated a SP disease course (Figure 2D). Several other strains presented with a monophasic disease course, which — in the case of CC068 — demonstrated a sex-specific staggered disease onset (Figure 1C and Figure 2E). Taken together, these results demonstrate that the genetic diversity in CC mice captures a wide spectrum of clinically relevant EAE phenotypes.

**Figure 3:**
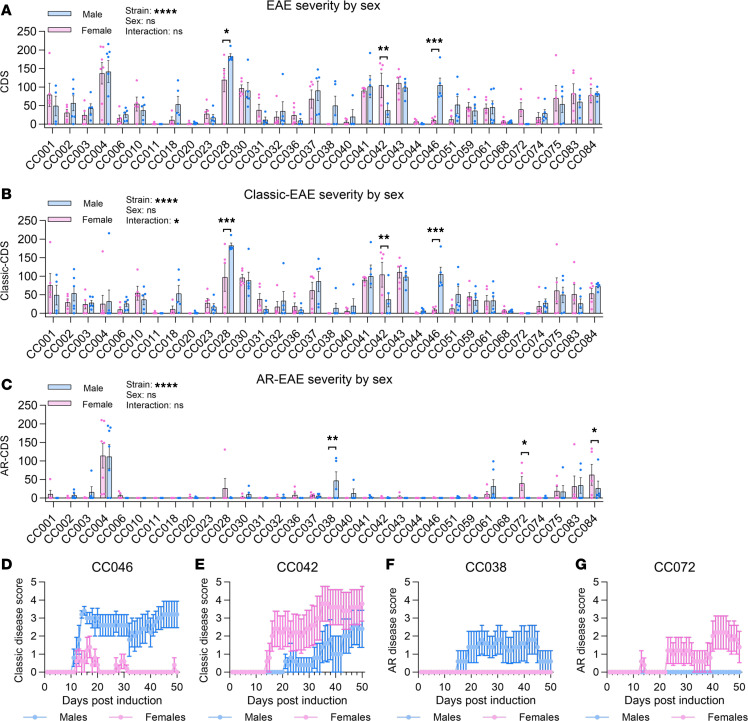
EAE in CC strains demonstrates bidirectional effects of sex on disease course. EAE was induced and observed in CC mice as described in Figure 1. (**A**–**C**) Disease severity was assessed for effects of sex within strain using CDS (**A**), classic-CDS (**B**), and AR-CDS (**C**). Significance of differences between sexes was determined by 2-way ANOVA with Fisher’s LSD multiple-comparison test (Supplemental Table 3). Comparisons are indicated by asterisks where significant. (**D**–**G**) Disease course profiles of sex differences in classic-EAE in CC046 (**D**) and CC042 (**E**), and AR-EAE in CC038 (**F**) and CC072 (**G**).

MS exhibits well-documented sex differences in both disease incidence and disease progression (6). Sex differences in EAE have also been reported, predominantly in SJL/J (SJL) mice and less so in B6 mice (29). With our sample size (~5/sex/CC strain), while we were likely underpowered to detect subtle sex differences within strains, we could capture larger effects if present. A 2-way ANOVA of the effect of strain and sex on CDS (analyzed separately for classic-EAE, AR-EAE, or combined disease) revealed a highly significant effect of strain (as expected), no overall effect of sex, and a significant strain by sex interaction in the case of classic-CDS (Figure 3, A–C). A post hoc analysis of the effect of sex within each CC strain identified significant and bidirectional effects of sex on EAE CDS in 6 strains across the different disease types (Figure 3, A–C; Table 1; and Supplemental Table 3). Examination of disease course revealed distinct differences for classic-EAE in CC046 (greater severity in males; Figure 3D) and CC042 (greater severity in females; Figure 3E), and for AR-EAE in CC038 (greater severity in males; Figure 3F) and CC072 (greater severity in females; Figure 3G). Because our initial experiments had low sample size when split by sex, we performed replicate experiments in 2 select CC strains. This confirmed our findings for CC042 (Supplemental Figure 9, A and B), while replicate experiments in CC018, a strain with initially suggestive sex differences (Supplemental Figure 5), revealed a significant difference in classic-EAE severity (greater in males) with increased sample size (Supplemental Figure 9, C and D). Taken together, these results demonstrate that the effect of sex on EAE is highly genotype dependent, indicating the presence of gene-by-sex interactions. Given the small sample size for the majority of the strains studied, these sex differences will need to be further validated in future studies.

### Distinct immunopathology in the spinal cord and brain is associated with severe classic and AR-EAE phenotypes.

**Figure 4:**
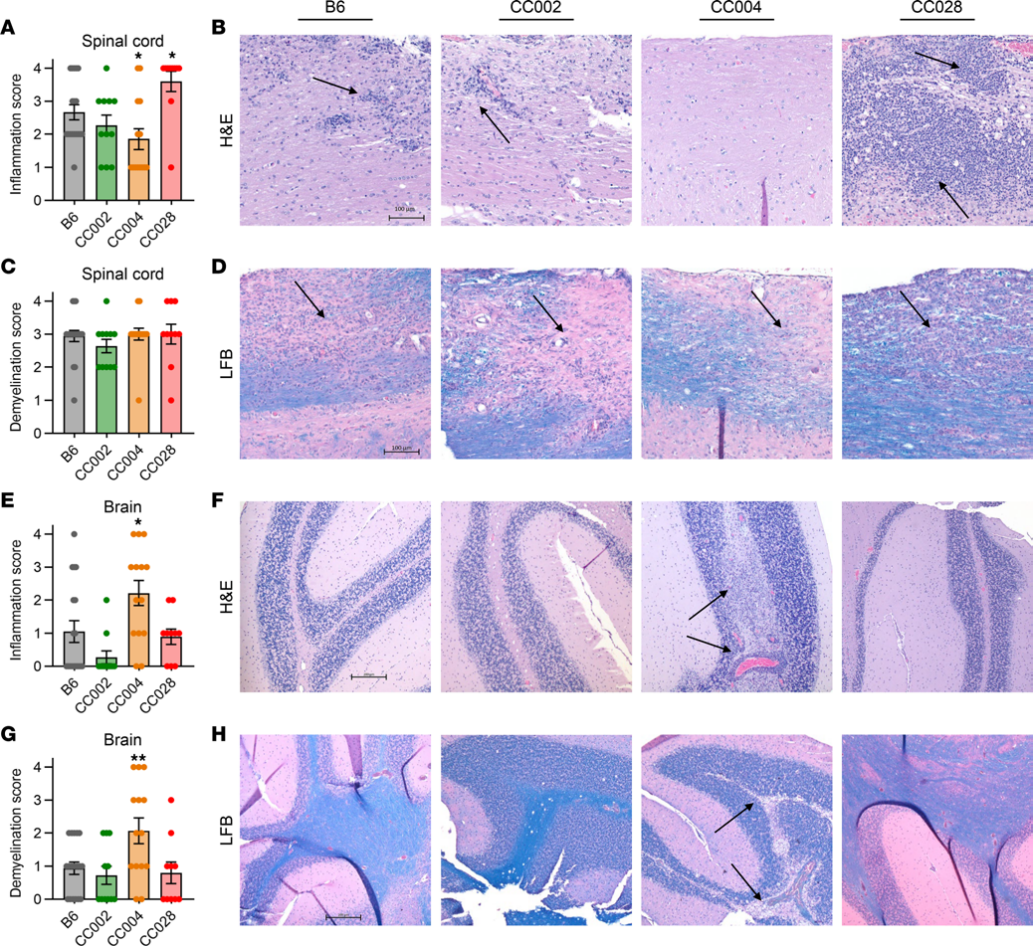
Severe progressive EAE in CC028 mice and AR-EAE in CC004 mice is associated with distinct pathology in the spinal cord and brain, respectively. EAE was induced and evaluated as described in Figure 1. On D50, or at humane endpoint, spinal cord and brains were collected and processed for staining with H&E with or without LFB. Histopathologic evaluation of B6 reference control (9M, 9F), CC002 (6M, 5M), CC004 (7M, 7F), and CC028 (5M, 5F) (sexes pooled) was performed as described in Methods. (**A** and **B**) Spinal cord inflammation scores by strain and corresponding representative images. (**C** and **D**) Spinal cord demyelination scores by strain and corresponding representative images. Spinal cord images (**B** and **D**) were captured at 10× objective. Scale bar: 100 μm. (**E** and **F**) Brain inflammation scores by strain and corresponding representative images. (**G** and **H**) Brain demyelination scores by strain and corresponding representative images. Brain images (**F** and **H**) were captured at 5× objective. Scale bar: 200 μm. For all images, the arrows mark regions of inflammatory infiltrates or demyelination. Significance of differences of each CC strain from B6 reference control was determined by ordinary 1-way ANOVA, with Fishers LSD multiple-comparison test (**A**, **C**, and **G**), or by Brown-Forsythe and Welch ANOVA, with unpaired *t* test with Welch’s correction for multiple comparison testing when appropriate (**E**). *P ≤ 0.05, **P ≤ 0.01.

To determine the immunopathological basis of the distinct EAE phenotypes, we specifically focused on strains exhibiting RR-EAE (CC002), AR-EAE (CC004), and severe-progressive EAE (CC028), together with B6 reference controls. In the experiments described above, brain and spinal cord tissues were collected at D50 (or humane endpoint), fixed in formalin, and processed for sectioning and staining with H&E or Luxol fast blue (LFB) to assess immune cell infiltration or demyelination, respectively. Analysis of spinal cord inflammation in B6 revealed expected focal inflammatory infiltrates, which were comparable in CC002 and were significantly reduced in CC004 mice (Figure 4, A and B). In contrast, spinal cords from CC028 mice demonstrated significantly greater levels of inflammation compared with B6 reference controls, characterized by extensive and dense infiltration of inflammatory cells (Figure 4, A and B). Surprisingly, the overall extent of demyelination in the spinal cord showed no significant difference between the 3 CC strains and B6 reference controls (Figure 4, C and D). Assessment of brain pathology revealed a CC004-specific increase in inflammation compared with B6 reference controls, characterized by prominent perivascular infiltrates and moderate-to-severe parenchymal infiltration in the cerebellum (Figure 4, E and F). Likewise, LFB-stained CC004 brain sections revealed a significant increase in the level of demyelination (Figure 4, G and H). Taken together, these data demonstrate that AR-EAE in CC004 mice is associated with lesions in the cerebellum rather than the spinal cord, consistent with previous findings (30), while severe-progressive classic-EAE in CC028 mice is associated with augmented spinal cord inflammation.

### Severe EAE in CC028 and CC004 is associated with a greater abundance of myeloid rather than lymphoid cells in the CNS.

**Figure 5:**
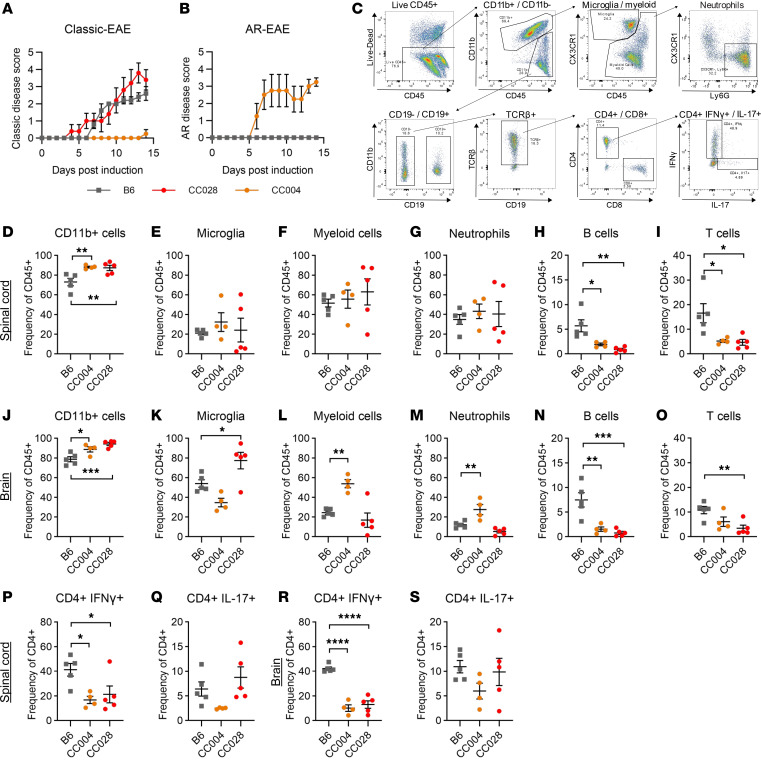
Severe EAE in CC028 and CC004 mice is associated with unique CNS immune profiles. EAE was induced in 8- to 14-week-old male B6 (*n* = 5), CC004 (*n* = 4), and CC028 (*n* = 5) mice as described in Figure 1. On D14, spinal cord and brains were collected and processed for flow cytometric staining. (**A** and **B**) Disease course profiles for B6, CC004, and CC028 mice displayed as classic-EAE or AR-EAE. (**C**) Representative gating scheme for flow cytometric analysis. (**D**–**I**) Scatter plots demonstrating frequencies of key immune cell subsets in the spinal cord of by strain, including CD11b^+^ cells (CD45^+^CD11b^+^) (**D**), microglial cells (CD45^int^CD11b^+^CX3CR1^+^) (**E**), myeloid cells (CD45^+^CD11b^+^Cx3CR1^lo/–^) (**F**), neutrophils (CD45^+^CD11b^+^CX3CR1^–^Ly6G^+^) (**G**), B cells (CD45^+^CD11b^–^CD19^+^) (**H**), and T cells (CD45^+^CD11b^–^CD19^–^TCRβ^+^) (**I**). (**J**–**O**) Frequencies of key immune cell subsets in the brain by strain, including CD11b^+^ cells (**J**), microglial cells (**K**), myeloid cells (**L**), neutrophils (**M**), B cells (**N**), and T cells (**O**). (**P** and **Q**) Frequencies of CD4^+^ T cells (CD45^+^CD11b^–^CD19^–^TCRβ^+^CD4^+^) producing IFN-γ (**P**) and IL-17 (**Q**) in the spinal cord by strain. (**R** and **S**) Frequencies of CD4^+^ T cells producing IFN-γ (**R**) and IL-17 (**S**) in the brain by strain. Significance of differences between each CC strain and B6 reference control was determined via 1-way ANOVA with Dunnett’s multiple-comparison test.*P ≤ 0.05, **P ≤ 0.01, ***P ≤ 0.001, ****P ≤ 0.0001.

To characterize immune infiltration in severe-progressive EAE (CC028) and AR-EAE (CC004) phenotypes, EAE was induced and followed until D14, to prevent mice from succumbing to humane endpoints, while capturing peak disease activity. D14 disease course recapitulated our results above (Figure 5, A and B). At D14, brain and spinal cord tissues were collected and processed independently for leukocyte isolation and flow cytometric analysis to assess key immune cell populations (Figure 5C). We found an increase in total CD11b^+^ cell frequency in both the brain and spinal cord of CC004 and CC028 compared with B6 mice (Figure 5, D and J). Further examination revealed no significant differences in microglia, myeloid cells, or neutrophils between the 3 strains in the spinal cord (Figure 5, E–G). However, analysis of these populations in the brain revealed a significantly greater frequency of microglia in CC028 compared with B6 mice (Figure 5K). Additionally, we found a significant increase in myeloid cells in the brain of CC004 compared with B6 mice (Figure 5L), which was mostly accounted for by an increase in neutrophils (Figure 5M). The increased frequencies of myeloid cells in the brain and spinal cord of CC004 and CC028 compared with B6 mice were counterbalanced by a lower frequency of B and T cells (Figure 5, H, I, N, and O). A similar pattern was seen for IFN-γ production by CD4^+^ T cells, which was reduced in CC004 and CC028 compared with B6 mice, in both tissues (Figure 5, P and R). This decrease in Th1 cells was not accompanied by a reciprocal change in Th17 cells, as evidenced by lack of significant differences in IL-17 expression in CD4^+^ T cells (Figure 5, Q and S). Taken together, these findings suggest that the severe-progressive classic-EAE phenotype of CC028 mice is surprisingly associated with a decrease in lymphocyte abundance in the spinal cord, including greatly reduced Th1 cells. While a similar decrease in lymphocytes is seen in the brain and spinal cord of AR-EAE CC004 mice, there is an additional brain-specific increase in neutrophil infiltration, which is in alignment with the brain-specific increase in inflammation and demyelination observed during histopathological analysis (Figure 4).

### RR-EAE in CC002 mice is driven by both peripheral immune responses and non–hematopoietic-derived factors.

**Figure 6:**
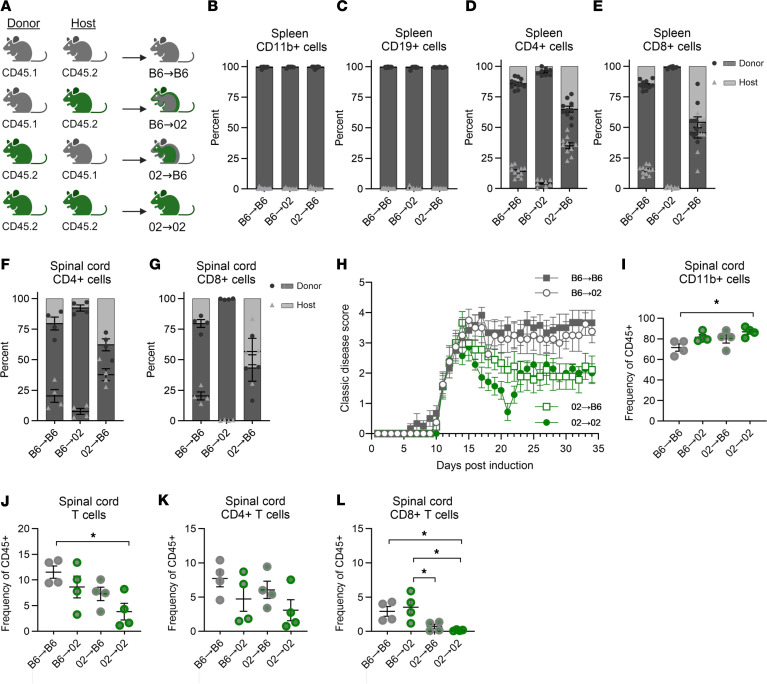
Peripheral immune and CNS intrinsic factors drive RR-EAE in CC002 mice. (**A**) B6 and CC002 mice were subjected to BM ablation and reconstitution to create reciprocal BM chimeric mice, designated as B6→B6 (7M, 4F), B6→02 (7M, 1F), 02→B6 (6M, 4F), 02→02 (6M, 1F) and illustrated in the schematic. Mice were rested for a total of 8 weeks prior to EAE induction as described in Figure 1. Mice were observed for a total of 34 days. On D34, spleen (B6→B6: 7M, 4F; B6→02: 7M, 1F; 02→B6: 6M, 4F; 02→02: 6M, 1F), and spinal cord (*n* = 4 males/chimera) tissues were collected and processed for flow cytometric staining. (**B**–**G**) Percent chimerism was assessed in B6→B6, B6→02, and 02→B6 for splenic CD11b^+^ cells (CD45^+^CD11b^+^CD19^–^) (**B**), CD19^+^ cells (CD45^+^CD11b^–^CD19^+^) (**C**), CD4^+^ T cells (CD45^+^CD11b^–^CD19^–^TCRβ^+^CD4^+^) (**D**), and CD8^+^ T cells (CD45^+^CD11b^–^CD19^–^TCRβ^+^CD8^+^) (**E**), as well as infiltrating CD4^+^ (**F**) and CD8^+^ (**G**) T cells in the spinal cord. Bars in **B**–**G** represent average percent chimerism for donor (dark gray) and host (light gray), while corresponding colored dots demonstrate individual samples. (**H**) Disease course profiles for B6→B6, B6→02, 02→B6, and 02→02 displayed as classic-EAE. (**I**–**K**) Comparison of spinal cord infiltrating immune cell populations in B6→B6, B6→02, 02→B6, and 02→02 for CD11b^+^ cells (**I**), CD19^+^ cells (**J**), CD4^+^ T cells (**K**), and CD8^+^ T cells (**L**). Significance of differences between groups was determined via 1-way ANOVA with Tukey’s multiple-comparison test and indicated by brackets and asterisks where significant. *P* ≤ 0.05.

While peripheral immune cells initiate disease in EAE/MS, CNS-intrinsic factors play an important role in regulating disease progression. To determine which of these 2 distinct mechanisms serve as the basis for the genetically regulated RR-EAE phenotype in CC002 mice, reciprocal BM chimera EAE experiments between MHC-matched (*H2^b^*) control B6 and CC002 mice were conducted. To assess chimerism, we used congenic B6 mice carrying the CD45.1 allele (B6.SJL-*Ptprc^a^Pepc^b^*/BoyJ), since CC002 mice carry an A/J-derived haplotype at the *Ptprc* locus, and therefore the CD45.2 allele; however for the CC002→CC002 chimeras congenic markers, could not be used (Figure 6A). At 8 weeks after BM ablation and reconstitution, EAE was induced as above and observed until D34, at which point the spleen and spinal cord were collected for immunophenotyping by flow cytometry.

Flow cytometric analysis using CD45.2 and CD45.1 markers demonstrated successful BM chimerism across all groups assessed, with some variation across cell types, as expected. An average of 95.4% (86.6%–99.4%) of total splenic leukocytes were donor derived. For CD11b^+^ and CD19^+^ cells, the average chimerism was greater than 98% (Figure 6, B and C). CD4^+^ and CD8^+^ T cells displayed an average of 81.2% and 78.2% chimerism, respectively, with increased persistence of B6 host T cells in CC002→B6 chimeras (Figure 6, D and E). Subsequent assessment of donor cell frequency in the spinal cord demonstrated similar trends to those observed in the spleen, most notably the persistence of some host CD4^+^ and CD8^+^ T cells in CC002→B6 chimeras (Figure 6, F and G).

Analysis of disease course demonstrated that the control group phenotypes matched our original findings (Figure 2C), with chronic-EAE observed in B6→B6 chimeras and RR-EAE in CC002→CC002 chimeras (Figure 6H), confirming that BM ablation and transplantation did not alter these phenotypes. Analysis of the experimental groups demonstrated that BM from B6 donors was sufficient to alter the disease course in CC002 hosts, resulting in a “B6-like” chronic-EAE phenotype (Figure 6H). Meanwhile, transfer of CC002 BM into B6 hosts resulted in remitting-EAE, although without relapse (Figure 6H). Assessment of CNS infiltrating cells revealed a significant difference between CC002→CC002 and B6→B6 chimeras, with a greater frequency of CD11b^+^ cells (Figure 6I) and a reduced T cell population (Figure 6J) in CC002→CC002 compared with B6→B6 chimeras. This T cell reduction was driven primarily by a significant decrease in frequency of CD8^+^ cells in strains with remitting (CC002→B6 and CC002→CC002) compared with chronic (B6→B6 and B6→CC002) EAE phenotypes (Figure 6L), an effect that was not observed in CD4^+^ cells (Figure 6K). Taken together, these results suggest that, while remission of EAE in CC002 mice is intrinsic to the peripheral immune system, CNS-intrinsic genetic factors may drive relapse. Alternatively, the lack of relapse in CC002→B6 chimeras could be driven by the significant percentage of remaining host (B6) T cells.

### QTL mapping reveals distinct loci associated with EAE subtype, course, and severity.

Besides identification of potentially novel phenotypes, an advantage of the CC model is the ability to map loci controlling phenotypic traits of interest. We performed genome-wide association mapping for 2 major EAE quantitative traits: (a) EAE incidence (Figure 1B), as a measure of disease susceptibility, and (b) CDS (Figure 1D), as a cumulative measure of disease severity, duration, and incidence. For incidence, we either measured total EAE incidence, or subsetted incidence by (a) disease subtype (classic and AR-EAE) and (b) disease course (chronic, RR, and monophasic). Association mapping was performed using R/qtl2 software (31), with genome-wide significance logarithm of odds (LOD) thresholds determined by permutation analysis, using a relaxed threshold (given the limited number of strains studied and the complexity of the traits) of 20% to identify top QTL. QTL were designated *Eaecc* (EAE QTL identified in CC mice) and numbered in the order of their description in the manuscript (*Eaecc1–6*).

**Figure 7:**
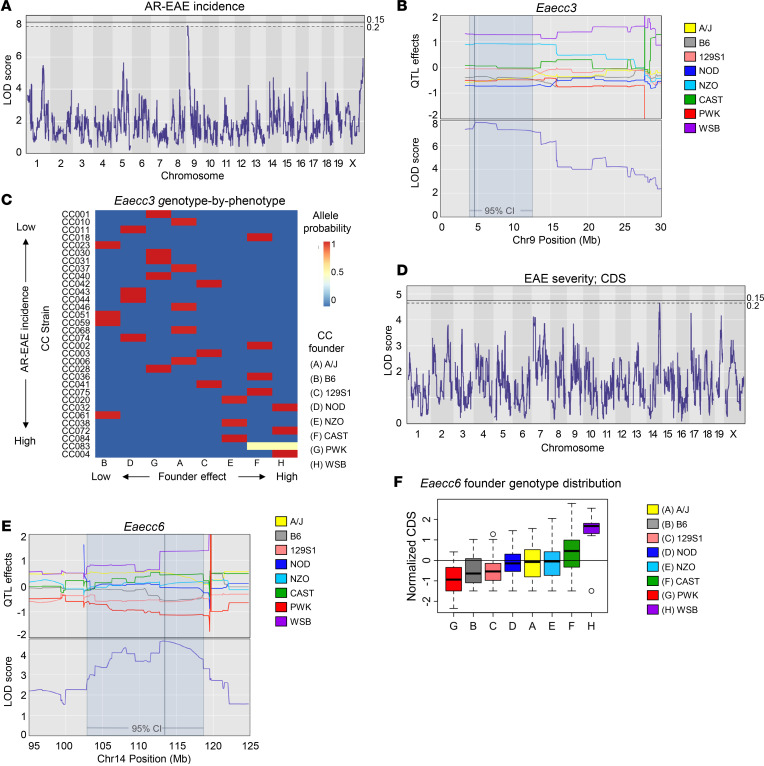
QTL analysis reveals distinct genetic linkage patterns for AR-EAE incidence and EAE severity. EAE was induced and evaluated in CC strains, as described in Figure 1. EAE QTVs were calculated, and QTL mapping was performed as described in Methods. (**A**) Manhattan plot demonstrating LOD traces for AR-EAE incidence, 15% and 20% genome-wide significance is indicated by the solid and dashed lines, respectively. (**B**) Corresponding CC founder allele effects plot for lead QTL on Chr9 — *Eaecc3*. (**C**) Heatmap demonstrating CC strain distribution based on genotype-by-phenotype analysis for *Eaecc3*. (**D**) Manhattan plot demonstrating LOD traces for EAE severity, 15% and 20% genome-wide significance is indicated by the solid and dashed lines, respectively. (**E**) Corresponding CC founder allele effects plot for lead QTL on Chr14 — *Eaecc6*. (**F**) Box and whisker plot (mean ± 95% CI) demonstrating distribution of CC founder alleles within strains.

The first trait mapped was total EAE incidence (any disease subtype). While no single QTL passed the significance threshold, the lead QTL on proximal chromosome (Chr) 4 (*Eaecc1*; LOD score = 6.79) fell just short of significance (Supplemental Figure 10, A and B, and Table 2). Analysis of classic-EAE incidence yielded a top QTL on Chr5 that fell short of significance (*Eaecc2;* LOD score = 6.64; Supplemental Figure 10, C and D, and Table 2). AR-EAE incidence mapping revealed a lead QTL on Chr9 (LOD score = 7.96) that passed 20% genome-wide significance (*Eaecc3;* Figure 7A and Table 2). Examination of founder effects revealed that WSB, and to a lesser extent NZO, alleles were associated with higher incidence of AR-EAE, while NOD alleles were associated with lower incidence (Figure 7B). Consistent with this, genotype-by-phenotype analysis revealed that, of the 32 studied CC strains, the top 5 CC strains with the highest AR-EAE incidence (CC004, CC083, CC084, CC072, and CC038) carried either WSB or NZO alleles at *Eaecc3* (Figure 7C). Subsetting EAE incidence by disease course revealed no major associations for chronic-EAE (Supplemental Figure 10E), but RR- and monophasic-EAE revealed 1 QTL each on Chr18 (*Eaecc4;* LOD score = 6.40) and Chr6 (*Eaecc5;* LOD score = 7.31), respectively, that fell just short of the significance threshold (Supplemental Figure 10, F–I, and Table 2).

Mapping of EAE severity (encompassing both classic and AR-EAE presentation) using CDS revealed a QTL on distal Chr14 passing 20% genome-wide significance (*Eaecc6*; LOD score = 4.65) (Figure 7D and Table 2). Examination of founder effects revealed that WSB alleles were associated with more severe EAE, while PWK alleles were protective (Figure 7, E and F). Taken together, these results reveal distinct linkage patterns for the incidence of different EAE subtypes, disease courses, and severity, suggesting that these phenotypes are controlled by several distinct major loci, further supporting the idea that disease course and severity in MS are genetically controlled.

### Candidate gene prioritization nominates candidate genes for QTL controlling EAE traits.

**Figure 8:**
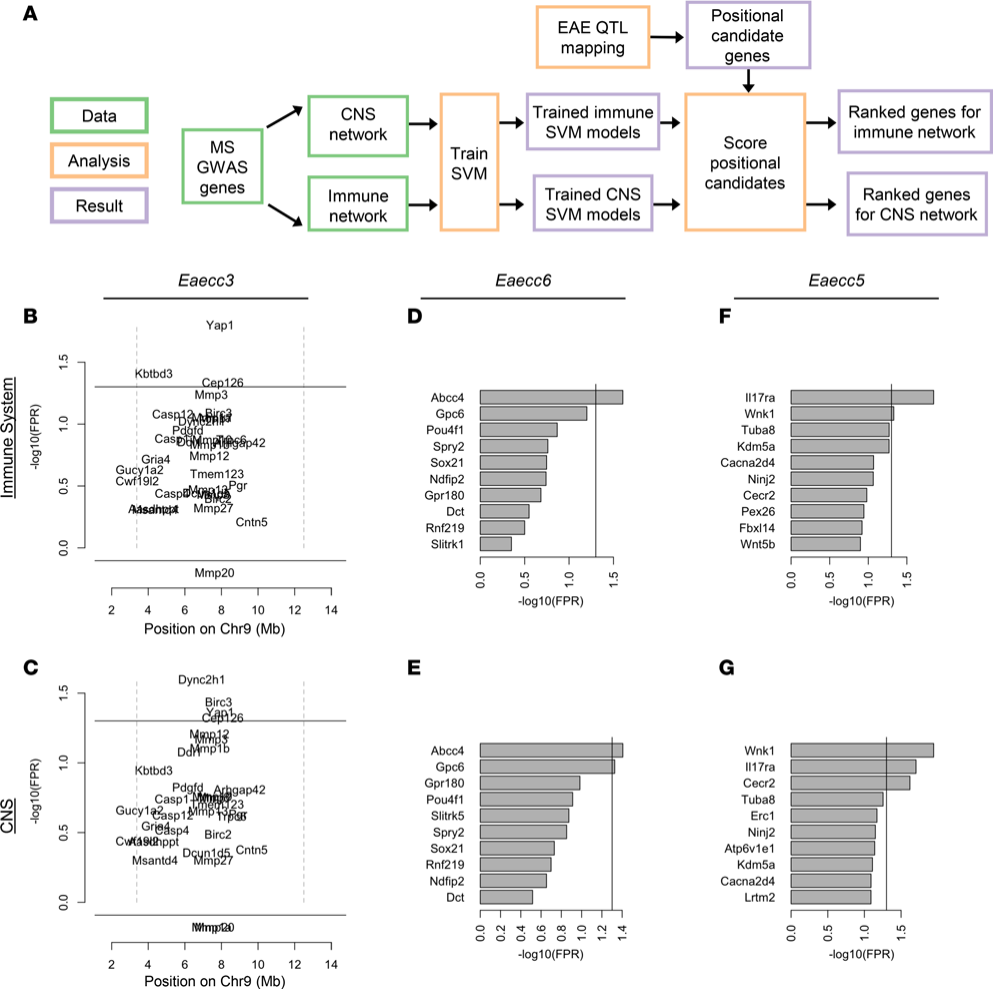
Machine learning–based functional candidate gene prioritization nominates distinct genes associated with QTL for AR-EAE incidence, EAE severity, and monophasic-EAE incidence. (**A**) SVM classifiers were trained using MS GWAS genes and integrated with tissue-specific connectivity networks to rank gene candidates associated with *Eaecc* QTL in the context of either the CNS or immune system, as illustrated by the schematic. (**B** and **C**) Ranked candidate genes for AR-EAE incidence (*Eaecc3*) in the immune system (**B**) and CNS (**C**). Genes are plotted by genomic position on the *x* axis and –log(FPR) on the *y* axis, dotted lines demonstrating QTL boundaries. (**D**–**G**) Ranked candidate genes, graphed independent of genomic position, for EAE severity (*Eaecc6*) in the immune system (**D**) and CNS (**E**), and monophasic-EAE incidence (*Eaecc5*) in the immune system (**F**) and CNS (**G**). The solid line in **B**–**G** corresponds an FPR threshold of 0.05.

The ultimate goal of genetic mapping is to identify the causative genes underlying the associated phenotypes of interest, which, in recombinant inbred strain populations like the CC, is often impeded by statistical resolution due to large haplotype blocks. This limitation can be overcome by gene prioritization approaches (32–34). Our prioritization analysis focused on the 2 QTL reaching 20% genome-wide significance: *Eaecc3* (Chr9; 95% CI, 4.04–11.82 Mb, ~9.1 Mb) and *Eaecc6* (Chr14; 95% CI, 103.97–118.63 Mb, ~14.7 Mb), as well as the suggestive narrow interval *Eaecc5* (Chr6; 95% CI, 118.97–121.49 Mb, ~2.5 Mb) (Table 2). While these QTL are moderately high resolution, these loci still contain numerous genes: *Eaecc3* (81 genes, 36 protein-coding), *Eaecc6* (130 genes, 19 protein-coding), and *Eaecc5* (68 genes, 32 protein-coding). To prioritize lead candidate genes in an unbiased manner, we utilized a machine learning–based approach that we developed previously (32–34) (Figure 8A). Briefly, support vector machine (SVM) classifiers were trained to distinguish trait-associated genes from randomly drawn genes using feature vectors derived from tissue-specific connectivity networks (35). The trained models were then asked to classify each positional candidate gene as trait related or not trait related. Here, we used the top 500 genes (by –log_10_[*P* value]) associated with MS from the National Human Genome Research Institute GWAS catalog (36) as the training set. We derived feature vectors for training from 2 tissue-specific mouse gene interaction networks: (a) the immune system, the initiator and driver of pathology in EAE/MS), and (b) the CNS, as the target organ in EAE/MS (Supplemental Data File 2).

For *Eaecc3* (AR-EAE incidence), this approach prioritized a total of 4 genes (2 immune system specific and 2 CNS specific) passing the false positive rate (FPR) cutoff of 0.05, with the 2 top prioritized genes being *Yap1* (immune system-specific) and *Dync2h1* (CNS-specific) (Figure 8, B and C, and Table 2). For *Eaecc6* (EAE severity), this approach prioritized 2 genes passing the FPR cutoff of 0.05: *Gpc6* (CNS-specific) and *Abcc4* (both tissues) (Figure 8, D and E, and Table 2). Additionally, *Gpc5*, adjacent to *Gpc6*, is itself an MS-GWAS candidate, although it was not ranked due to insufficient connectivity in the networks. Assessment of *Eaecc5* (monophasic-EAE incidence) prioritized a total of 3 genes (1 CNS specific and 2 in both tissues), with the top prioritized genes identified as *Il17ra* and *Wnk1* (Figure 8, F and G, and Table 2). Gene prioritization for the remaining suggestive QTL, *Eaecc1*, *Eaecc2*, and *Eaecc4*, identified additional lead candidate genes, including *Tox*, *Klf3*, and *Fbn2*, respectively (Supplemental Figure 11 and Table 2). Taken together, this analysis prioritizes several genes as plausible candidates driving the EAE phenotypes of interest via effects on the immune system or the CNS.

Because missense variants have a high potential effect on gene function, we identified nonsynonymous single nucleotide variants (nsSNPs) distinguishing the founder alleles exerting the strongest opposing effects at each of the 3 lead QTL (Figure 7, B and D, and Supplemental Figure 10G), focusing only on the top prioritized candidate genes. Assessment of nsSNPs segregating between WSB and NOD at the top 2 prioritized genes (*Yap1* and *Dync2h1*) for *Eaecc3* found no divergent nsSNPs. However, analysis of the remaining genes passing the 0.05 FPR cutoff revealed 1 nsSNP in *Birc3* (Table 3). For *Eaecc6*, comparison of nsSNPs differentiating WSB from PWK in the top 2 prioritized genes revealed 1 nsSNP in *Gpc6*, and none in *Abcc4* (Table 3). For *Eaecc5*, comparison of PWK and 129S1 alleles in *Il17ra* revealed no segregating nsSNPs, while 2 nsSNPs were identified in *Wnk1* (Table 3). Taken together, these results, combined with our prioritization analysis, highlight potential coding variants driving EAE phenotypes of interest, to be functionally validated in future studies.

## Discussion

The major risk genes for MS incidence reside in the MHC locus (7, 9). While we originally intended to use the full set of 7 MHC haplotypes represented across the CC population, this was precluded by the low efficacy of the “MHC-agnostic” EAE induction using mSCH (Supplemental Figure 1). While we found no significant effect of the 2 selected haplotypes (*H2^b^* and *H2^g7^*) on EAE (Figure 1, E–K, and Supplemental Figure 8), this does not rule out a role of additional MHC alleles in disease progression, which has been suggested in a several human studies (28, 37). This can be addressed in future studies utilizing recombinant encephalitogenic proteins or intercrossing *H2*-compatible and noncompatible CC strains.

Historically, distinct EAE disease profiles have been reported utilizing varying myelin-derived peptides for EAE induction in corresponding susceptible strains of mice (25). For example, EAE induced using proteolipid protein peptide 135–151 (PLP_135–151_) in SJL (*H2^s^*) mice has been commonly used to model RR-EAE (25). AR-EAE was documented in PLP_109–209_–immunized C3H/HeJ mice (*H2^k^*), accompanied by distinct cerebellum or brain stem pathology (30). Later studies suggested that AR-EAE was associated with higher Th17/Th1 skewing and increased neutrophil infiltration (38–41). Our results of brain-specific pathology in AR-EAE–presenting CC004 mice (Figure 3) are in line with these findings. However, while we also found a brain-specific increase in neutrophil infiltration and a reduction in CD4^+^ IFN-γ production in CC004 mice, we did not find a corresponding increase in IL-17 (Figure 4). Other historic models include SP disease in MOG_35–55_–induced NOD mice (26) and mSCH-induced EAE in Biozzi ABH mice (42), although their relevance to progressive MS is debated (43). Importantly, with the methods presented here, we were able to model a variety of distinct EAE disease profiles, including RR disease (CC002) and CIS-like monophasic disease (CC068), using a common peptide-induction approach. While we identified 2 CC strains with either rapidly progressive severe disease (CC028) or secondary progression following a remission (CC043), more studies are needed to determine whether these represent valid models of progressive MS.

The SJL EAE model is commonly used to model sex differences in MS (6). In a previous mapping study using an F2 intercross between SJL and B10.S mice, half of the identified EAE loci showed sex-specific bias (44). In the CC, we saw no overall sex effect and identified 6 of 32 strains with sex bias. It is possible that the use of a B6-oriented EAE induction protocol or the lack of SJL-derived alleles in the CC results in moderate sex effects.

Given the availability of extensive GWAS assessing MS incidence, we leveraged these results for comparison with *Eaecc* QTL. Candidate gene prioritization of *Eaecc2* (classic-EAE incidence) identified 2 genes, *Klf3* and *Rhoh* (Supplemental Figure 11, C and D), both of which have been associated with MS risk (7). Furthermore, comparison of all identified genes within *Eeaecc* QTL (without prioritization) to MS incidence–associated genes (7, 45) revealed additional overlapping genes, including: *Txk/TXK* and *N4bp2/N4BP2* (*Eaecc2), Chd7/CHD7* and *Ints8/INTS8* (*Eaecc1,* EAE incidence), and *Gpc5/GPC5* (*Eaecc6,* EAE severity). Taken together, these results identify overlapping genes driving predominantly disease incidence/susceptibility, rather than disease course, in both EAE and MS, supporting a shared genetic architecture between the human disease and its model, in concordance with our previous EAE QTL mapping studies in SJL and B10.S mice (46).

With regard to our candidate gene prioritization approach, we note as a caveat that the candidates may be somewhat biased towards those associated with disease risk/susceptibility rather than progression. Our approach requires a large training set of “true positive” phenotype-associated genes (Supplemental Data File 2). The majority of these genes (available from the GWAS catalog) were associated with MS risk/incidence, although a small number represented emerging GWAS hits for MS progression/severity (discussed below). Therefore, our prioritized candidate genes are more closely associated with MS genes linked to susceptibility, rather than progression.

While there is a wealth of genetic associations with MS risk/incidence, GWAS of disease severity/progression are just beginning to emerge (23, 24, 47, 48). We used these studies to generate a list of genes associated with MS severity/progression (Supplemental Data File 2) and assessed overlap with *Eaecc* QTL. This identified *Spry2/SPRY2* in *Eaecc6* (EAE severity), which is the gene nearest to rs2876767, a suggestive hit from a GWAS analysis of MS severity (23). Our comparison also revealed *Chd17/CHD17* (MS severity; ref. 24) and *Ppargc1a/PPARGC1A* (accumulation of disability in MS; ref. 47), overlapping with *Eaecc1* and *Eaecc2*, respectively. Taken together, these studies further highlight the value of using the CC model to map genes associated with MS progression as an orthogonal approach to resource-intensive GWAS in humans. In particular, mouse QTL studies could prioritize/refine the mapping of the candidate genes from human GWAS, as in the example of *Spry2/SPRY2*, above.

Beyond overlapping MS GWAS genes, candidate gene prioritization for promising *Eaecc* QTL revealed genes relevant to MS pathology. Assessment of *Eaecc6* (EAE severity) identified *Abcc4/ABCC4,* encoding the ATP-binding cassette transporter ABCC4. Relevant to MS pathology, ABCC4 has been implicated in blood-brain barrier function (49) and effector immune cell efflux (50). *Yap1/YAP1,* a key component of the Hippo signaling pathway (51), and *Dync2h1/DYNC2H1*, which encodes a dynein motor protein involved in intraciliary transport, were the top prioritized genes for *Eaecc3* (AR incidence). Recent studies have suggested the involvement of the Hippo pathway in autoimmunity (52), with emphasis on Treg and proinflammatory Th17 cell differentiation through *Yap*-*Taz* expression (51). While less direct evidence for involvement in EAE/MS pathogenesis exists for *Dync2h1/DYNC2H1*, this gene has been implicated in neurodevelopment, maintenance, neuronal transport (53–55), and retinopathies (56), suggesting a potential involvement in neurodegeneration. Analysis of *Eaecc5* (monophasic-EAE) prioritized *Il17ra/IL17RA*, encoding the receptor for the proinflammatory cytokines IL-17A/F, and *Wnk1/WNK1,* encoding a serine/threonine protein kinase involved in CNS signaling. The association of *Il17ra/IL17RA* with monophasic-EAE incidence is plausible, given the involvement of IL-17 and Th17 cells in MS/EAE pathogenesis (57, 58), particularly CIS and early MS (59, 60). Similarly, *Wnk1/WNK1* is involved in pathogenic signaling in the CNS (61) and has been implicated as a crucial component in neuronal axon development and maintenance (62), representing a potential mechanism for association with monophasic-EAE incidence.

To our knowledge, our study is the first to apply the CC resource in an autoimmune model of MS. However, the Threadgill and Brinkmeyer-Langford groups have utilized the CC to study neurological disease induced by Theiler’s murine encephalitis virus (63–67). While these studies were likely underpowered for QTL mapping and complicated by the fact that host genetic variation affects viral clearance in this model, they provide strong evidence that neurological sequelae following immune-mediated demyelination in the CNS are genetically regulated, in agreement with our own EAE studies.

Our identification and initial characterization of EAE phenotypes in CC mice represents what we hope will be the first of many utilizing the CC resource in the pursuit of the genetic underpinnings behind MS disease heterogeneity. An obvious extension will include the validation and functional characterization of the identified candidate genes, as well as further mapping efforts to increase the resolution and statistical significance of *Eaecc* QTL. Other efforts will focus on mechanistic characterization of unique EAE phenotypes. Importantly, the work presented here provides to the research community an easily accessible animal model of MS that accounts for host genetic diversity and captures phenotypes lacking in traditional models.

## Methods

### Sex as a biological variable.

Approximately equal numbers of male and female mice were utilized for EAE phenotypic screening and whenever possible for follow-up experiments. Due to availability limitations, male mice were utilized for flow cytometric analysis. However, based on the alignment of EAE phenotypes between sexes in the strains used for said experiments, the results derived are expected to be relevant for both sexes.

### Mice.

For peptide-based EAE studies, male and female mice of each of the 32 CC strains (Table 1) were purchased between 2021 and 2022 from the Mutant Mouse Resource and Research Center (MMRRC) at UNC, an NIH-funded strain repository, in collaboration with the UNC Systems Genetics Core Facility, with the exception of CC020, which was purchased from The Jackson Laboratory (Jax). While CC strains were originally generated and bred at Oak Ridge National Laboratory, the International Livestock Research Institute/Tel Aviv University, or Geniad Ltd., all CC strains had been maintained at UNC (or transferred from UNC to Jax and then back to UNC for clean rederivation) for 10 years from these original locations (68–71). All CC strains are referred to by their abbreviated name (“CC###”); full strain names are provided in Table 1. Mice were obtained as 4 cohorts — denoted as C1–C4 (Supplemental Data File 1). Male and female B6 mice were purchased from Jax as reference controls for each cohort. Once at the vivarium at UVM, mice were rested for a range of 14–29 days, prior to experimentation. For follow up studies utilizing CC002, CC004, and CC028, three females and 2 males of each strain were purchased from the MMRRC in collaboration with the UNC Systems Genetics Core Facility in 2022 and bred at the vivarium at UVM to generate offspring for experimentation.

### Selection of CC strains for peptide-induced EAE.

Founder strain contributions at the *H2* locus, specifically in the region encompassing MHC class I (*H2^K^* only) and all class II genes (Chr17:33918830–34347345), were identified using the UNC Systems Genetics Collaborative Cross Viewer tool.

### EAE induction and scoring.

mSCH was prepared from Swiss Webster mice (Charles River Laboratories), and EAE was induced in CC and CC founder strains using a modified approach from previously described methods (16, 18). Briefly, mice were injected s.c. with 0.15 mL of an emulsion containing 5 mg mSCH in PBS and 50% CFA (Sigma-Aldrich), supplemented with 4 mg/mL heat-killed *Mycobacterium tuberculosis* (Difco Laboratories). On D0 or D0 and D2, mice received 200 ng PTX (List Biological Laboratories) i.p. Mice were scored daily starting at D7 as described below.

For MOG_35–55_ EAE, mice were injected s.c. with 200 μg MOG_35–55_ (New England Peptide) emulsified in CFA, supplemented with 4 mg/mL heat-killed *M. tuberculosis*, and were treated with a single i.p. injection of 200 ng PTX on D0. Mice were scored daily for a total of 50 days starting at D5.

Mice were assigned an EAE score based on the presence of clinical disease symptoms, using both classic and a modified AR-EAE scoring scale (17, 27). Briefly, classic-EAE clinical scores were assigned as follows: 0, asymptomatic; 1, tail paralysis; 2, tail paralysis and hind limb weakness; 3, hind limb paralysis; 4, hind limb paralysis with incontinence; and 5, moribund/quadriplegic. AR-EAE clinical scores were assigned as follows: 0, asymptomatic; 1, slight head tilt; 2, pronounced head tilt; 3, inability to walk in a straight line; 4 mouse is moving/lying on its side, will continuously fall to its side after being made to stand; 5, mouse rolls or spins continuously. Mice reached humane endpoints after presenting with a score of 5 (classic or AR-EAE) for 72 hours, at which point mice were euthanized and their daily disease score was recorded as a 5 (classic or AR-EAE) for the remainder of the experiment. Similarly, mice that died after having presented with EAE for more than 2 consecutive days were given a daily disease score of 5 (classic or AR-EAE) for the remainder of the experiment. Mice that died without any EAE clinical signs or having presented with EAE for ≤ 2 days were excluded.

### EAE disease phenotype and QTV classification.

Raw daily disease scores, reported as both classic and AR-EAE, were utilized to derive the following daily score classifications: disease score (reports disease scores regardless of EAE subtype and derives an average score for any occurrence of simultaneous classic and AR-EAE scoring), classic disease score (reports disease scores that correspond only to classic-EAE), and AR disease score (reports disease scores that correspond only to the AR-EAE) (Supplemental Data File 1). Disease score classifications were utilized to determine CDS (the total sum of all daily disease scores), classic-CDS (the total sum of all daily classic disease scores), and AR-CDS (the total sum of all daily AR disease scores).

To assess incidence of EAE and EAE subtypes, the following QTVs were calculated: EAE incidence, classic-EAE incidence, and AR-EAE incidence. EAE incidence was determined utilizing daily disease scores and classified as ≥ 2 consecutive days of a score > 0. Classic-EAE incidence and AR-EAE incidence were assessed in a binary and mutually exclusive manner, as determined by raw daily disease score. First, mice were categorized into 3 groups: classic-EAE only, AR-EAE only, and “combined type” (mice that presented with both the classic and AR-EAE either on the same or different days). Mice that were classified classic-EAE only or AR-EAE only were designated as classic- or AR-EAE incidence, respectively. For mice classified as “combined type,” EAE subtype incidence was assigned as classic-EAE incidence if the number of days scored as classic-EAE (excluding simultaneous classic and AR-EAE) was greater than the number of days scored as AR-EAE (excluding simultaneous classic and AR-EAE). The inverse was utilized to assign AR-EAE incidence within “combined type.”

To determine EAE disease course, individual mice were classified as having either a monophasic-, RR-, or chronic-EAE. Monophasic-EAE was defined by a disease score > 0 for ≥ 2 consecutive days followed by remission to a score of zero for the remainder of the experiment. RR-EAE was defined as an initial disease (≥ 2 consecutive days of score > 0), followed by remission (score of 0 for ≥ 3 consecutive days) and a relapse (≥ 2 consecutive days of score > 0). Chronic-EAE was defined as persistent disease scores > 0 from time of disease onset until experiment termination that could not be classified as either monophasic or RR-EAE.

### CNS histopathology.

On D50 after EAE induction, or upon humane endpoint, mice were euthanized, and brain and spinal cord tissues were collected for histopathological assessment as previously described (72). Briefly, the skull and vertebral column were removed and diffusion fixed in 10% neutral buffered formalin (Thermo Fisher Scientific). After fixation, brain and spinal cord were extracted from calvaria and vertebral columns, respectively, and dissected into thirds (brain: coronal orientation front brain, mid brain, and hind brain — including cerebellum and brain stem; spinal cord: cervical, thoracic, and lumbar). Tissues were subsequently embedded in paraffin, sectioned (coronal and longitudinal for brain and spinal cord, respectively), and stained with LFB and/or H&E.

Histopathological assessment was conducted in a blinded fashion. Regions were assessed for degree of inflammation and demyelination using a semiquantitative scale adapted from previous studies (72). Inflammation was evaluated using H&E-stained tissues and scored as follows: 0, no inflammation; 1, few inflammatory cells scattered/small clusters; 2, organized clusters of inflammatory cells without significant extension beyond small lesions; 3, significant organized clusters of inflammatory cells with patchy infiltration of surrounding tissue, central involvement of larger lesions; and 4, extensive and dense infiltration of inflammatory cells affecting over half of the tissue. Extent of demyelination was evaluated using tissues stained with LFB + H&E and scored as follows: 0, no demyelination deep blue staining; 1, small, patchy area(s) of white matter pallor, no well-defined lesions; 2, defined area of white matter pallor forming isolated lesion(s); 3, confluent foci of white matter pallor with some spared areas; and 4, widespread white matter pallor affecting over ~75% of the tissue. Inflammation and demyelination scores were assigned to each of the 3 regions for both brain and spinal cord. Overall inflammation and demyelination scores were reported per mouse for brain and spinal cord separately, determined as the highest scored region of that tissue.

### Flow cytometry.

Mice were anesthetized under isoflurane and perfused transcardially with PBS, and brain and spinal cord tissues were collected and processed independently for flow cytometric staining as previously described (73). Tissues were mechanically dissociated to create a single-cell suspension, which was filtered and processed through Percoll gradient (37%/70%) centrifugation (390*g*) for leukocyte isolation. For intracellular cytokine analysis, cells were stimulated with 5 ng/mL PMA, 250 ng/mL ionomycin, and brefeldin A (BD Bioscience) for 4 hours prior to staining. Cells were then stained with LIVE/DEAD stain (Invitrogen) and then surface stained, followed by fixation, permeabilization and intracellular staining. Antibodies used are in Supplemental Table 4.

For flow cytometry staining to assess chimerism in addition to CNS infiltrating cells, spleens and spinal cords were collected. Spinal cord tissues were processed as described above, and spleens were processed as previously described (73). Cells were surface stained as above but included the CD45.1 and CD45.2 markers (Supplemental Table 4).

All stained cells were analyzed utilizing a Cytek Aurora and SpectroFlo software versions 2.2–3.3 (Cytek Biosciences). Spectral unmixing was performed with appropriate single-color controls and autofluorescence correction from an unstained control group. Data analysis was performed using FlowJo software versions 10.8.1–10.10 (BD Biosciences).

### Reciprocal BM chimeras.

CD45.2^+^ allele status of CC002 mice was determined using founder strain contributions for the gene *Ptprc* (Chr1:137990599-138103446 bp) using the UNC Systems Genetics Collaborative Cross Viewer tool. Reciprocal BM chimeras were generated between B6.SJL-Ptprc^a^Pepc^b^/BoyJ (B6.CD45.1; Jax) and CC002 mice as previously described (73). Nine- to 15-week-old recipient mice were irradiated twice with 550 rads 4–6 hours apart. After irradiation, mice were injected via retroorbital vein with 4 × 10^6^ whole BM cells from unmanipulated age- and sex-matched donors. Resulting chimeric mice were rested for 8 weeks to allow for maximal immune reconstitution, at which point EAE was induced.

### QTL mapping.

To map genetic variants associated with EAE QTV (including CDS and EAE incidence by disease subtype and course), the R package, R/qtl2 (31), was utilized. QTVs were calculated as described above. For QTL mapping with CDS, data from individual mice were utilized and underwent covariate batch correction, using experimental batch (cohort) as a covariate, and rank *Z* normalization. For QTL mapping of incidence QTVs, per-strain incidence percentages were utilized, and rank *Z* normalization was performed. CC genotype probabilities and kinship matrices were derived utilizing the CC genome sequenced data set available from the UNC Systems Genetics Core Facility (http://csbio.unc.edu/CCstatus/CCGenomes/#genotypes). Thresholds for 15% and 20% genome-wide significance were generated utilizing 1,000 permutations.

### Candidate gene prioritization.

SVMs were trained to classify randomly selected genes from previously identified MS GWAS genes as reported by the National Human Genome Research Institute GWAS catalog (36). The top 500 genes by –log_10_(*P* value) were utilized for training (Supplemental Data File 2). Mouse orthologs were identified for training set genes, and positional candidates were removed so as not to be used for SVM training. Feature vectors for training SVMs were based on connection weights in 1 of 2 functional networks of tissues in mouse network (35) — hemolymphoid system, as a proxy for the immune system, or CNS. The feature vector for a single true positive gene consisted of its connection weights to all other true positive genes in the training set. Any genes without connections were trimmed off, resulting in a total of 271 and 273 positive-labeled genes for training the immune system and CNS networks, respectively (Supplemental Data File 2). For each tissue-specific network, we trained 100 independent SVMs. Each SVM was trained to classify positively labeled MS genes from a matched set of randomly drawn non-MS GWAS genes. All trained SVMs were then used to classify positional candidate genes in each QTL. The final score for each gene was the –log_10_ of the FPR averaged across all 100 SVMs. FPR for gene *x* was defined as follows: FPR_x_ = FP/(FP + TN), where FP is the number of false positive genes and TN is the number of true negative genes using a cutoff score equal to that of gene *x*. Significance for prioritization analysis was determined using an FPR of 0.05.

To determine direct overlap between the identified candidate genes associated with *Eaecc* QTL and mouse orthologs of genes associated with MS risk, we compared all genes within a given *Eaecc* QTL to the complete list of reported genes from the 2019 GWAS analysis (7) as well as the genes associated with MS susceptibility from the original list of the top 500 genes used in the SVM training pipeline. Similarly, we generated a list of mouse orthologs of genes associated with MS severity/progression based on recent studies (23, 24, 47, 48) (Supplemental Data File 2) to determine overlap between the identified candidate genes associated with *Eaecc* QTL and genes associated with MS severity.

### Statistics.

Statistical analysis not pertaining to QTL mapping and candidate gene prioritization was carried out using GraphPad Prism software, versions 9.1.2–10.2.1. Assessment of effects between B6 and CC strains were determined by ordinary 1-way ANOVA, with Dunnett’s, Tukey’s, or Fishers LSD multiple-comparison tests, or by Brown-Forsythe and Welch ANOVA, with unpaired *t* test using Welch’s correction for multiple-comparison testing, when appropriate. Assessment of within-strain sex effects were determined by 2-way ANOVA with Fisher’s LSD or Šídák’s multiple-comparison tests. Analysis of effects of H2 haplotype were determined via 2-tailed unpaired *t* test. Details of the analyses are provided in the figure legends, including the specific tests used and adjustments for multiple comparisons when appropriate. Comparisons were assessed for effects between B6 and CC strains or within strain sex effects as indicated. All center values represent the mean, and data are shown as mean ± SEM. *P* < 0.05 was considered significant.

### Study approval.

The experimental procedures used in this study were approved by the UVM IACUC, protocol no. X2-034.

### Data availability.

Values for all data points in graphs are reported in the Supporting Data Values file.

## Author contributions

DNK, EAN, JMM, MTF, RML, and CT designed the research; EAN, ALT, TLW, KGL, and KCH performed the research; EAN, ALT, MTF, JMM, and DNK analyzed the data and interpreted results; EAN and DNK wrote the manuscript; and EAN, DNK, ALT, TLW, MTF, RML, and CT edited the final manuscript.

## Supplementary Material

Supplemental data

Supplemental data set 1

Supplemental data set 2

Supporting data values

## Figures and Tables

**Table 1:**
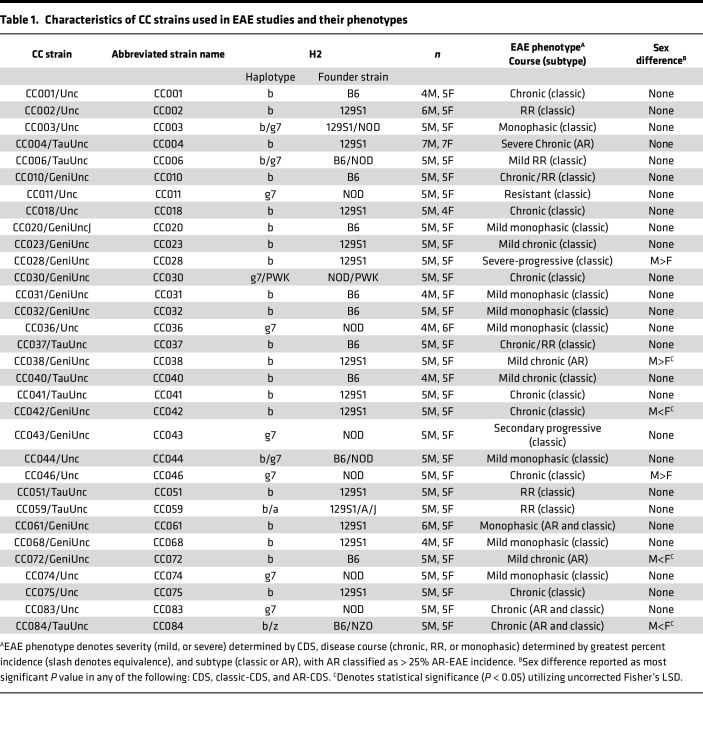
Characteristics of CC strains used in EAE studies and their phenotypes

**Table 2:**
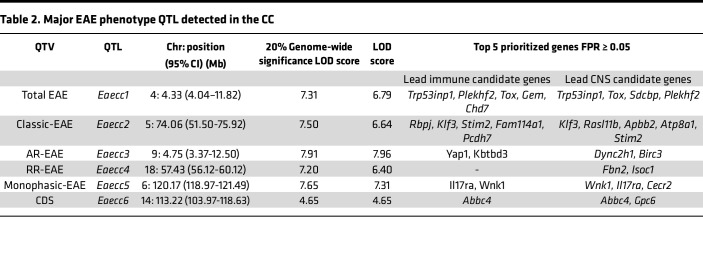
Major EAE phenotype QTL detected in the CC

**Table 3:**
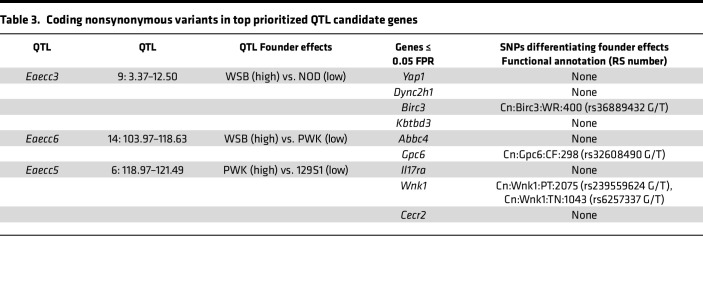
Coding nonsynonymous variants in top prioritized QTL candidate genes

## Version 1 09/26/2024

In-Press Preview

## Version 2 11/08/2024

Electronic publication
