# Supplementary material for: Analysis of CNS autoimmunity in genetically diverse mice reveals unique phenotypes and mechanisms
Source: JCI Insight. 2024 Nov 8;9(21):e184138. doi: 10.1172/jci.insight.184138 (PMC11601571; doi:10.1172/jci.insight.184138)
Supplement: Supplemental data [file jciinsight-9-184138-s116.pdf]

Supplemental Figure 1

A

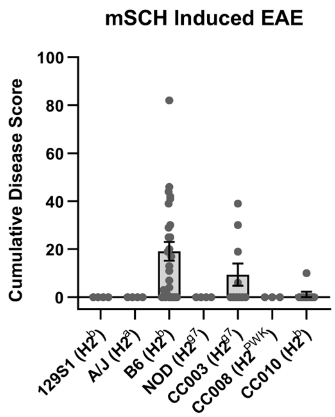

Supplemental Figure 1. mSCH-based induction of EAE in CC mice with various *H2* haplotypes is inefficient. EAE was induced 8-20 week old mice with various *H2* haplotypes including: CC founder strains - 129S1 ($H2^b$; 4M), A/J ($H2^a$; 4M), B6 ($H2^b$; 17M + 10F), and NOD ($H2^{g7}$; 4M), and CC strains: CC003 ($H2^{g7}$;8M + 2F), CC008 ($H2^{PWK}$; 3F), and CC010 ($H2^b$; 2M + 7F) by s.c. immunization with 0.15 ml of an emulsion containing 5 mg mSCH in PBS and 50% CFA. On D0 or D0 and D2, mice were administered an i.p. injection of 200 ng PTX as an ancillary adjuvant (see Methods). Mice were observed daily for a total of 26 days starting at 7 days post induction for the presence of clinical disease symptoms. (**A**) Cumulative disease score of above-mentioned strains shown with bars to demonstrate strain averages and points to display individual mice.

Supplemental Figure 2: 1 of 2

EAE Disease Course – Strain Average

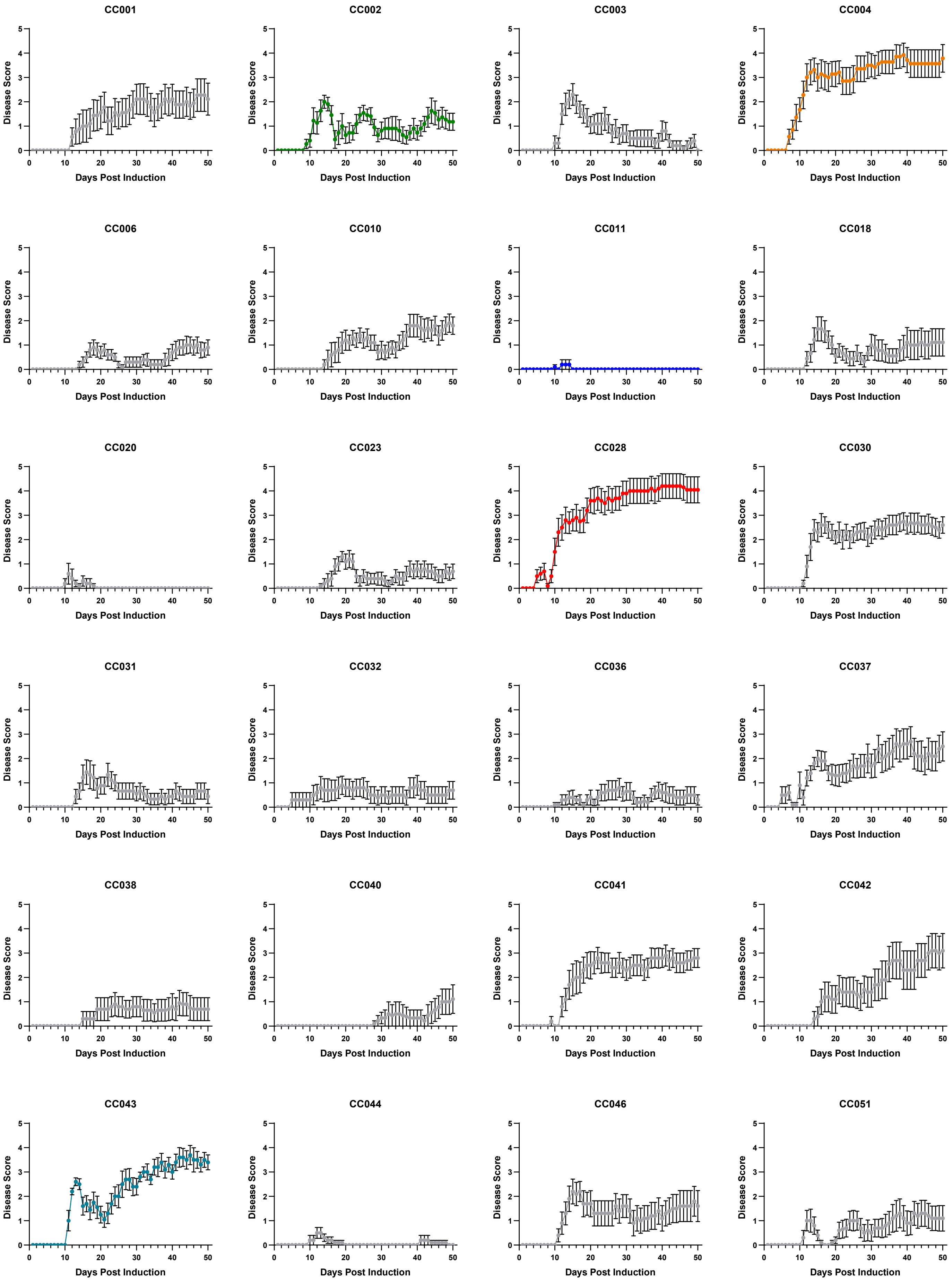

Supplemental Figure 2: 2 of 2

EAE Disease Course – Strain Average

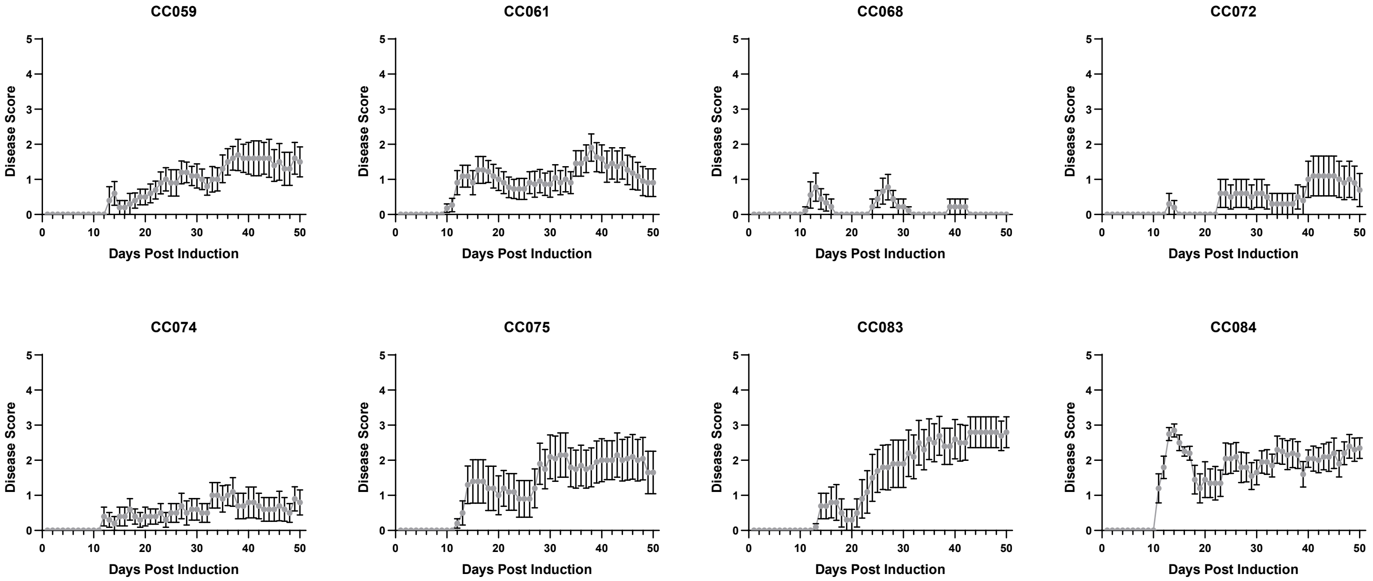

Supplemental Figure 2. Disease course profiles for CC strains. EAE was induced and observed for 50 days in CC mice as described in Figure 1. Disease course profiles for each strain were derived from daily disease scores (combined scores for both classic and AR-EAE; see Methods). Disease course profiles as calculated by strain average for each CC strain are displayed. CC strains are displayed in numerical order by strain number and strains highlighted in Figure 2 retained strain specific label coloring.

Supplemental Figure 3: 1 of 2

EAE Disease Course – Sexes

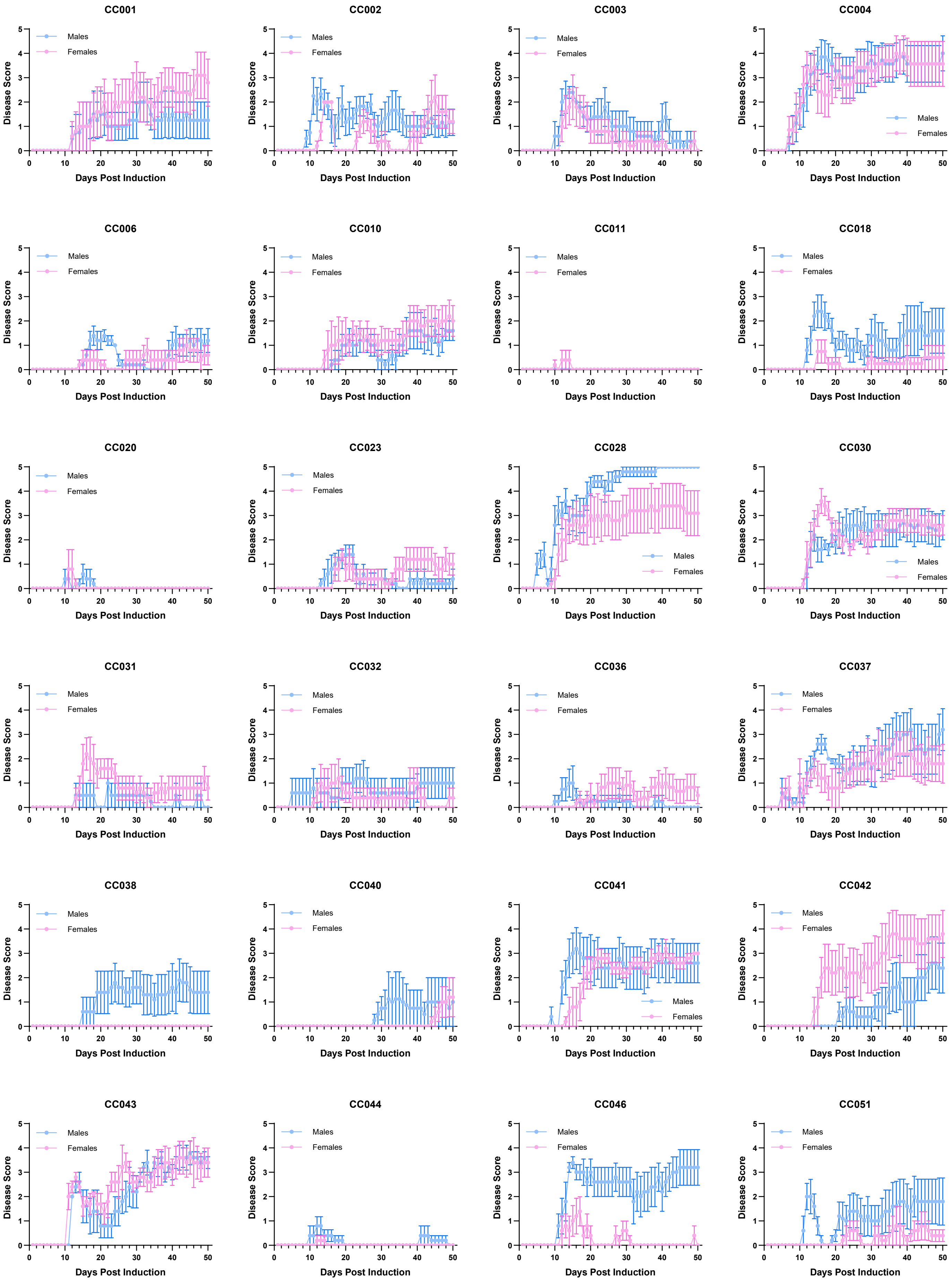

Supplemental Figure 3: 2 of 2

EAE Disease Course – Sexes

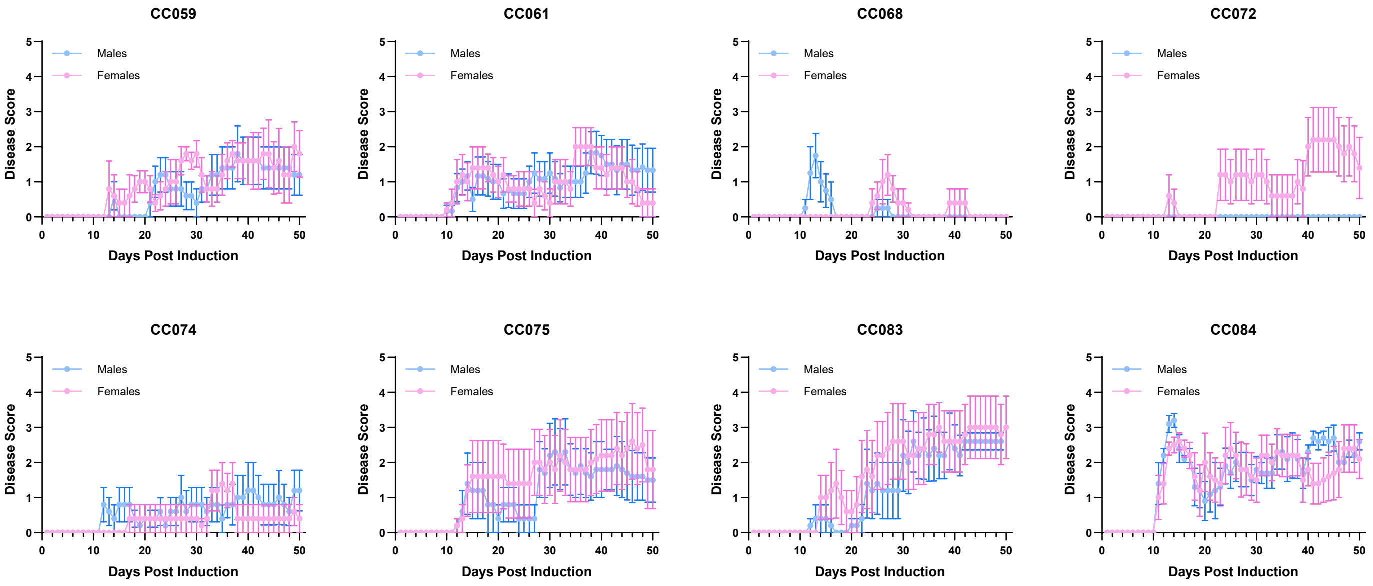

Supplemental Figure 3. Disease course profiles for CC strains separated by sex. EAE was induced and observed for 50 days in CC mice as described in Figure 1. Disease course profiles for each strain were derived from daily disease scores (combined scores for both classic and AR-EAE; see Methods). Sex specific disease course profiles as calculated by male (blue) and female (pink) averages for each CC strain are displayed. CC strains are displayed in numerical order by strain number.

Supplemental Figure 4: 1 of 2

Classic-EAE Disease Course – Strain Average

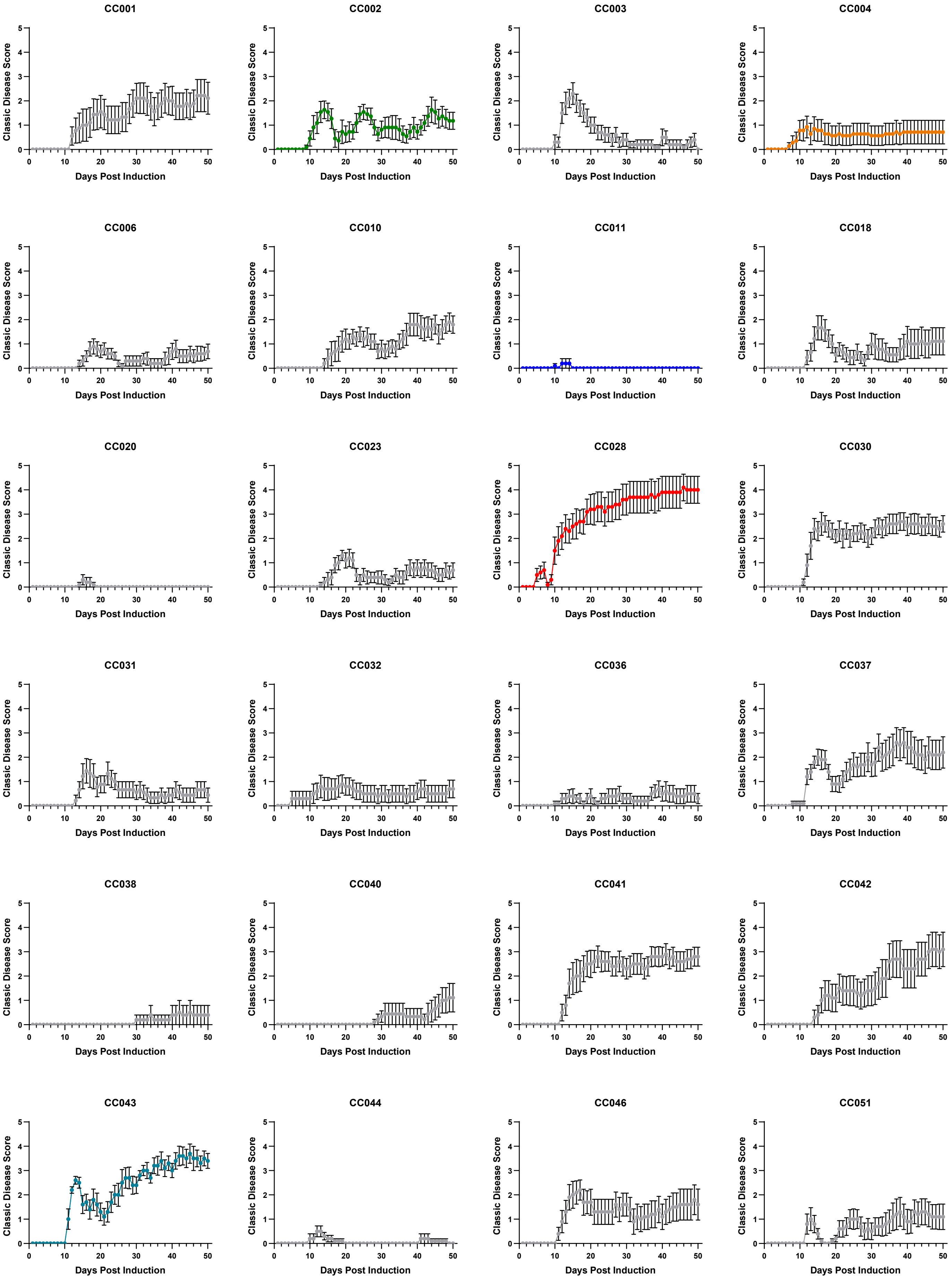

Supplemental Figure 4: 2 of 2

Classic-EAE Disease Course – Strain Average

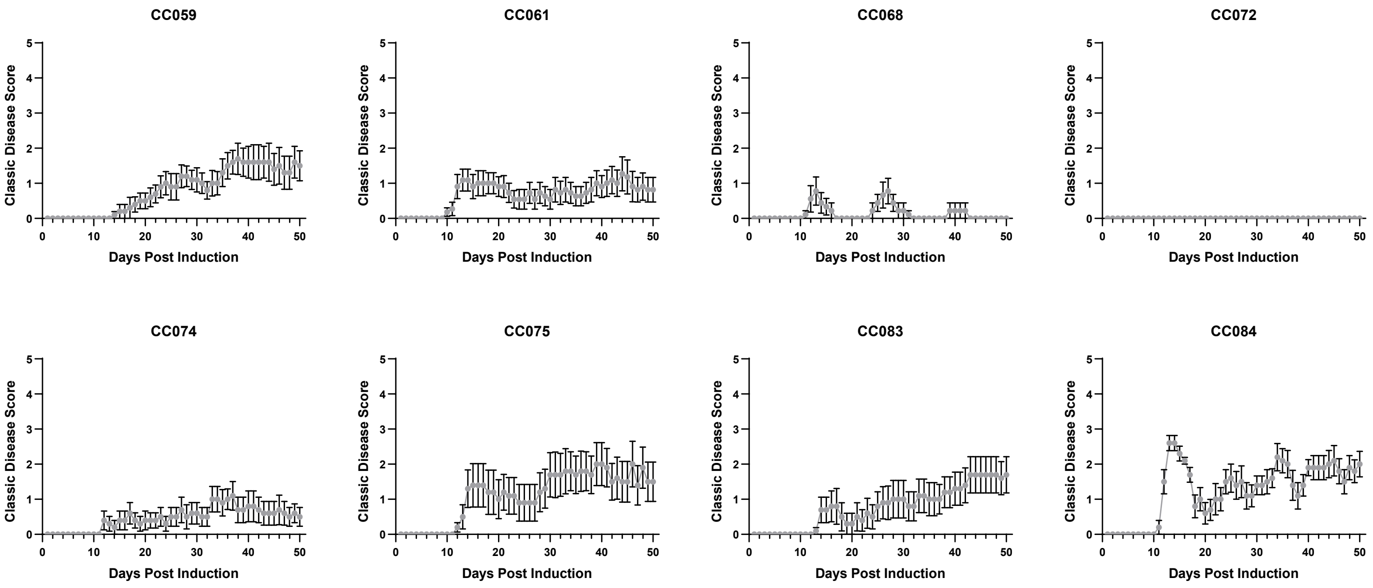

Supplemental Figure 4. Classic-EAE disease course profiles for CC strains. EAE was induced and observed for 50 days in CC mice as described in Figure 1. Classic-EAE disease course profiles for each strain were derived from daily classic-EAE disease scores (see Methods). Classic-EAE disease course profiles as calculated by strain average for each CC strain are displayed. CC strains are displayed in numerical order by strain number and strains highlighted in Figure 2 retained strain specific label coloring.

Supplemental Figure 5: 1 of 2

Classic-EAE Disease Course – Sexes

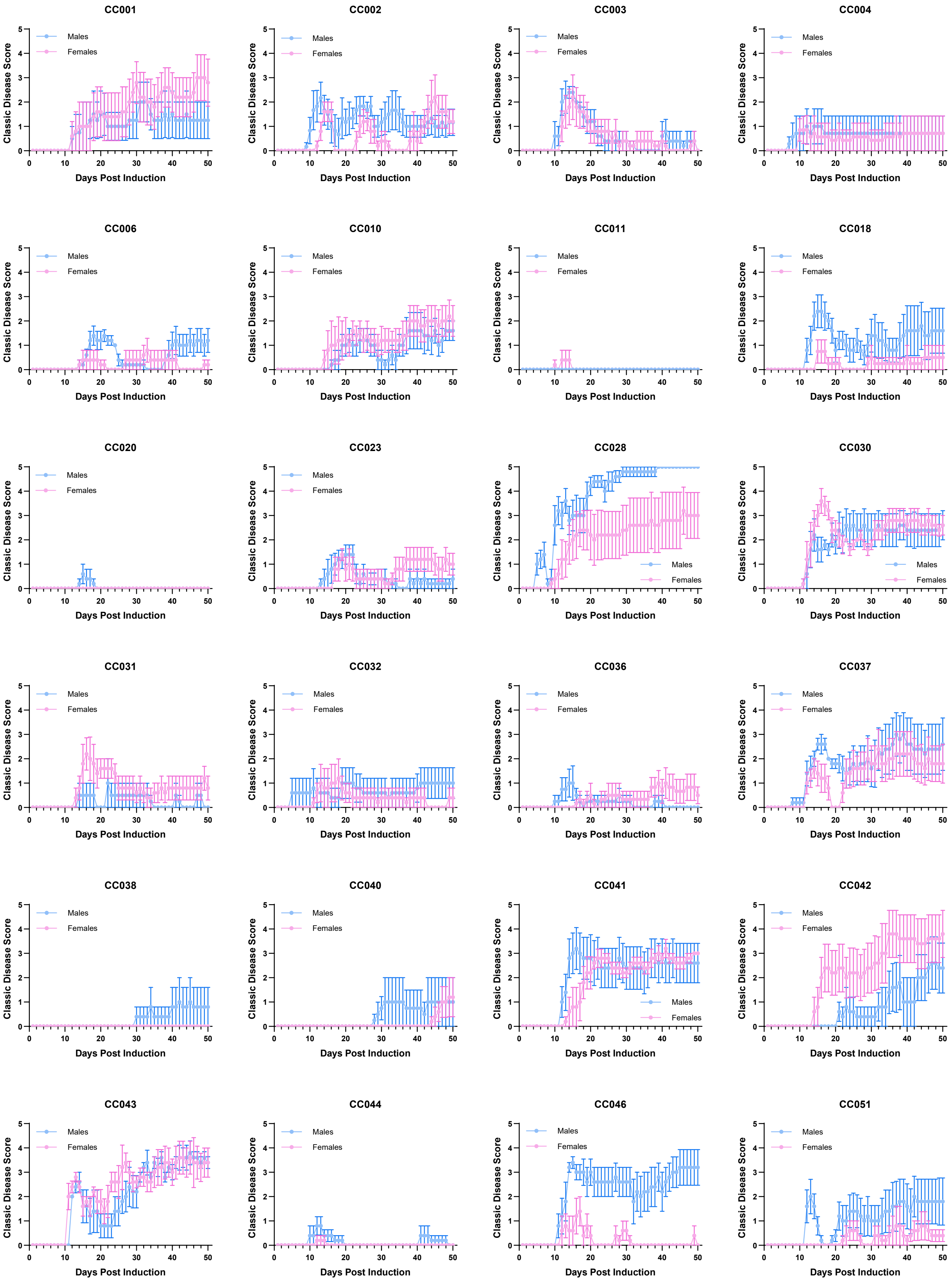

Supplemental Figure 5: 2 of 2

Classic-EAE Disease Course – Sexes

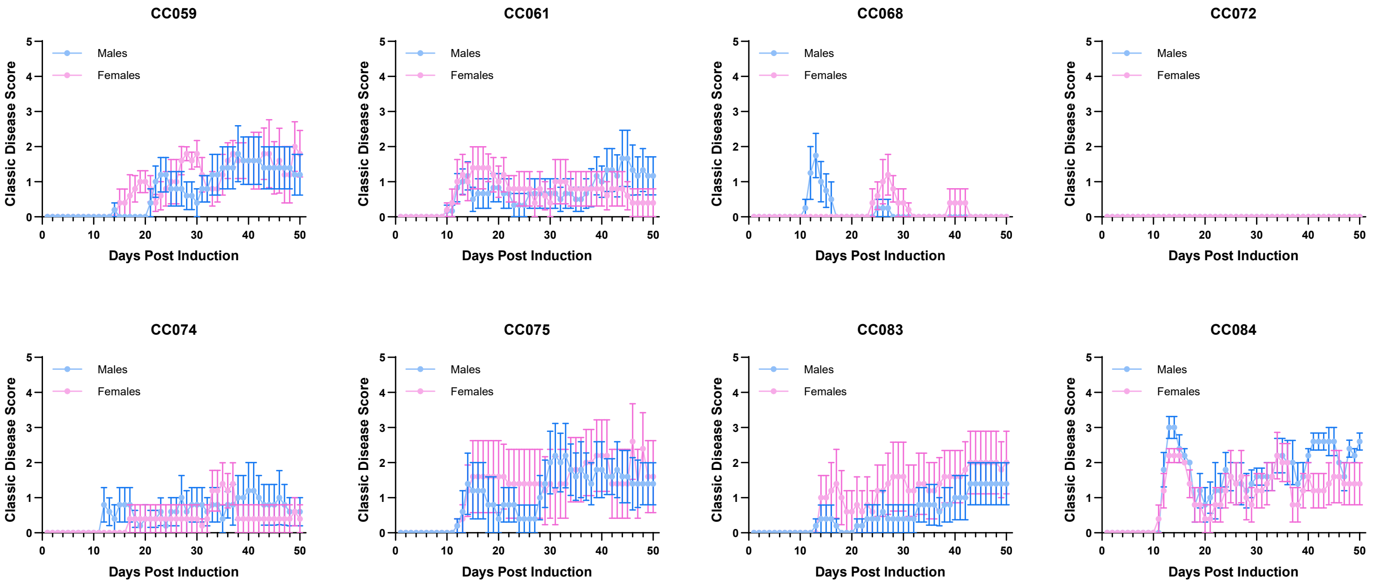

Supplemental Figure 5. Classic-EAE disease course profiles for CC strains separated by sex. EAE was induced and observed for 50 days in CC mice as described in Figure 1. Classic-EAE disease course profiles for each strain were derived from daily classic EAE disease scores (see Methods). Sex specific classic-EAE disease course profiles as calculated by male (blue) and female (pink) averages for each CC strain are displayed. CC strains are displayed in numerical order by strain number.

Supplemental Figure 6: 1 of 2

Axial Rotary -(AR) EAE Disease Course – Strain Average

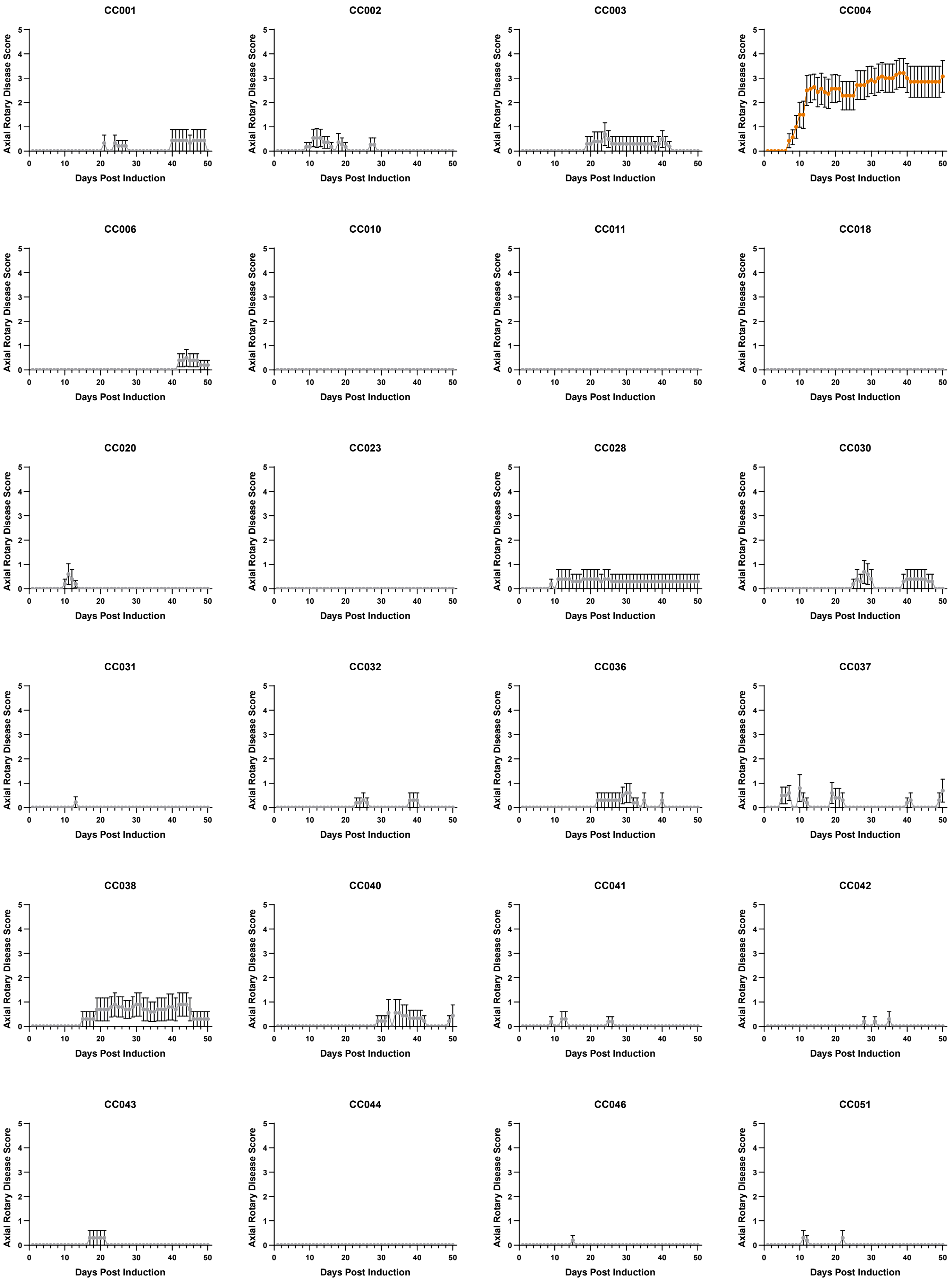

Supplemental Figure 6: 2 of 2

Axial Rotary -(AR) EAE Disease Course – Strain Average

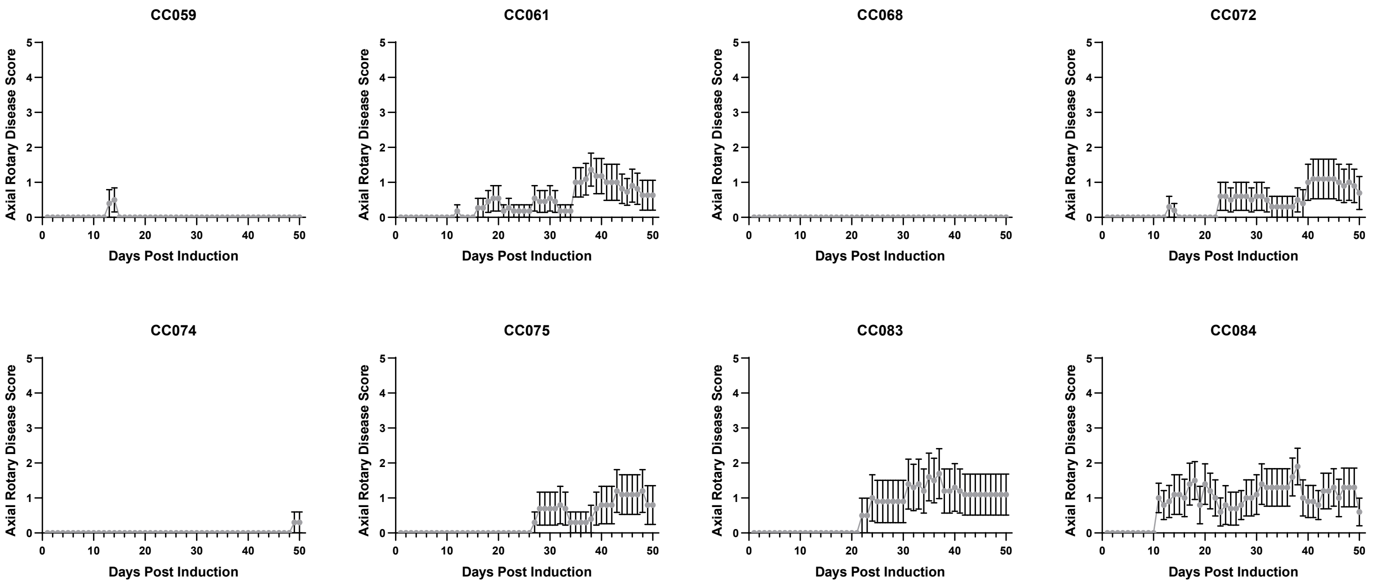

Supplemental Figure 6. AR-EAE disease course profiles for CC strains. EAE was induced and observed for 50 days in CC mice as described in Figure 1. AR-EAE disease course profiles for each strain were derived from daily AR-EAE disease scores (see Methods). AR-EAE disease course profiles as calculated by strain average for each CC strain are displayed. CC strains are displayed in numerical order by strain number and strains highlighted in Figure 2 retained strain specific label coloring.

Supplemental Figure 7: 1 of 2

Axial Rotary -(AR) EAE Disease Course – Sexes

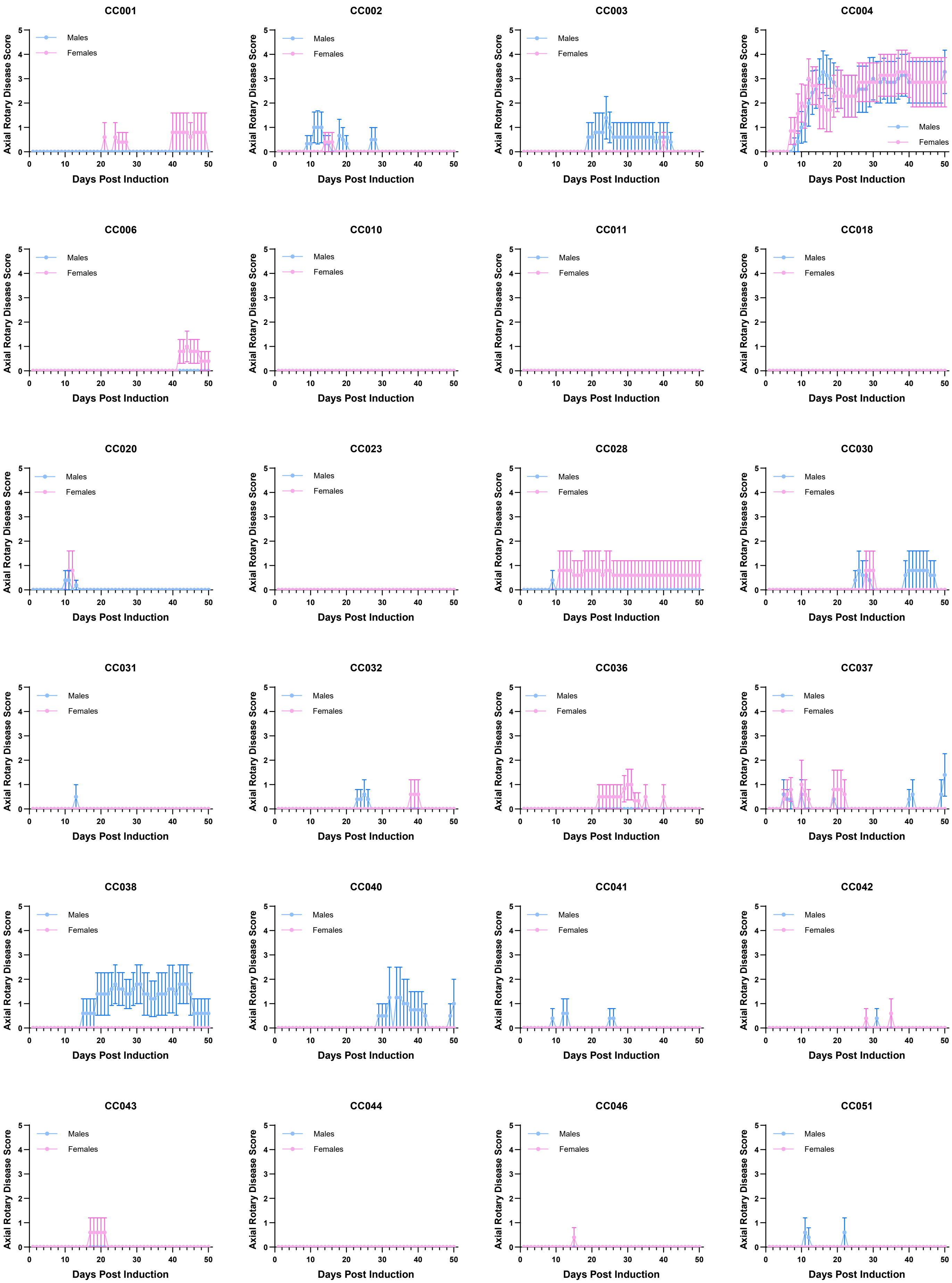

Supplemental Figure 7: 2 of 2

Axial Rotary -(AR) EAE Disease Course – Sexes

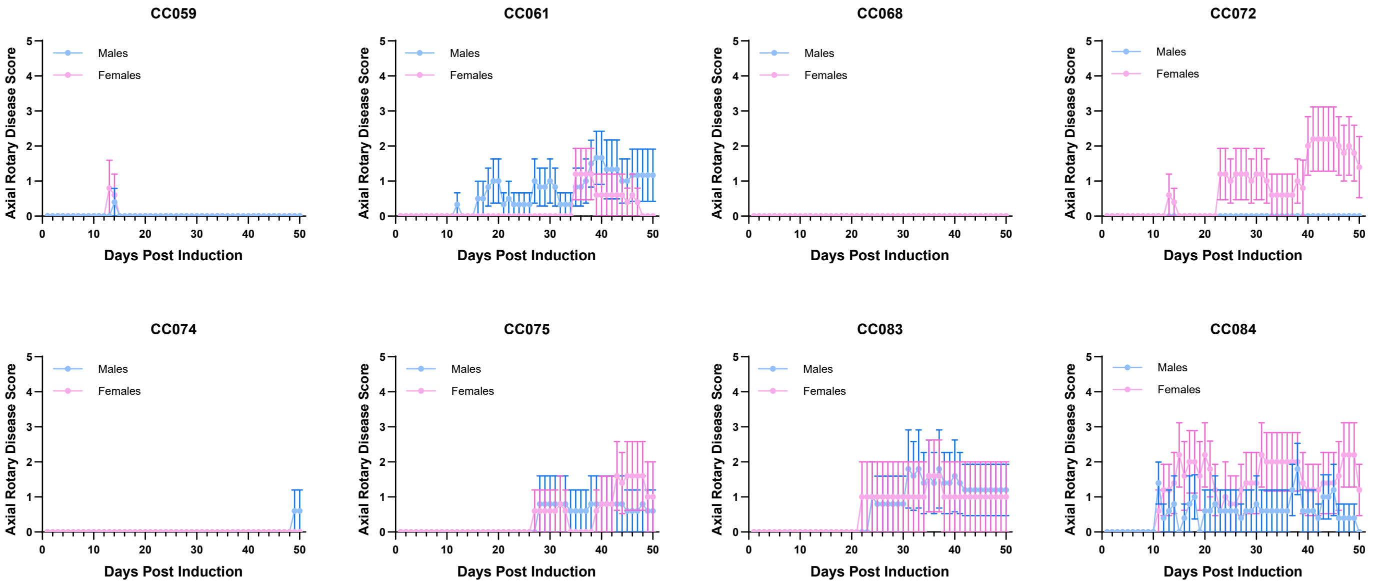

Supplemental Figure 7. AR-EAE disease course profiles for CC strains separated by sex. EAE was induced and observed for 50 days in CC mice as described in Figure 1. AR-EAE disease course profiles for each strain were derived from daily AR-EAE disease scores (see Methods). Sex specific AR-EAE disease course profiles as calculated by male (blue) and female (pink) averages for each CC strain are displayed. CC strains are displayed in numerical order by strain number.

Supplemental Figure 8

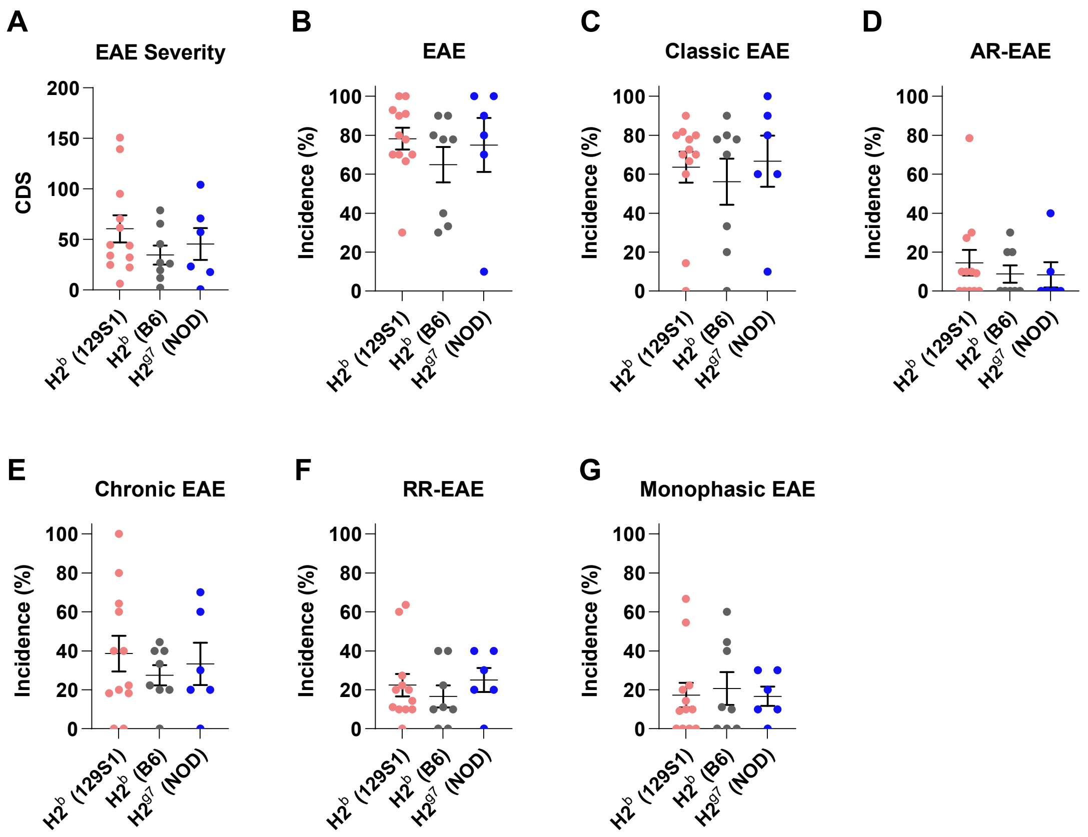

Supplemental Figure 8. EAE disease phenotypes are independent of CC founder specific *H2* effects. EAE was induced and observed for 50 days in CC mice as described in Figure 1. The indicated disease phenotypes were quantified (see Methods) and the potential impact of CC founder *H2* was assessed (**A-G**). Distribution of strain (**A**) CDS, (**B**) total EAE incidence, and incidence of (**C**) classic-EAE, (**D**) AR-EAE, (**E**) chronic-EAE, (**F**) RR-EAE, and (**G**) monophasic-EAE, grouped by founder derived $H2^b$ and $H2^{g7}$ homozygous haplotypes. Each data point represents an average for a single CC strain. Significance of differences between haplotypes was determined by one-way ANOVA with Tukey's multiple comparisons test and indicated by asterisks where significant.

Supplemental Figure 9

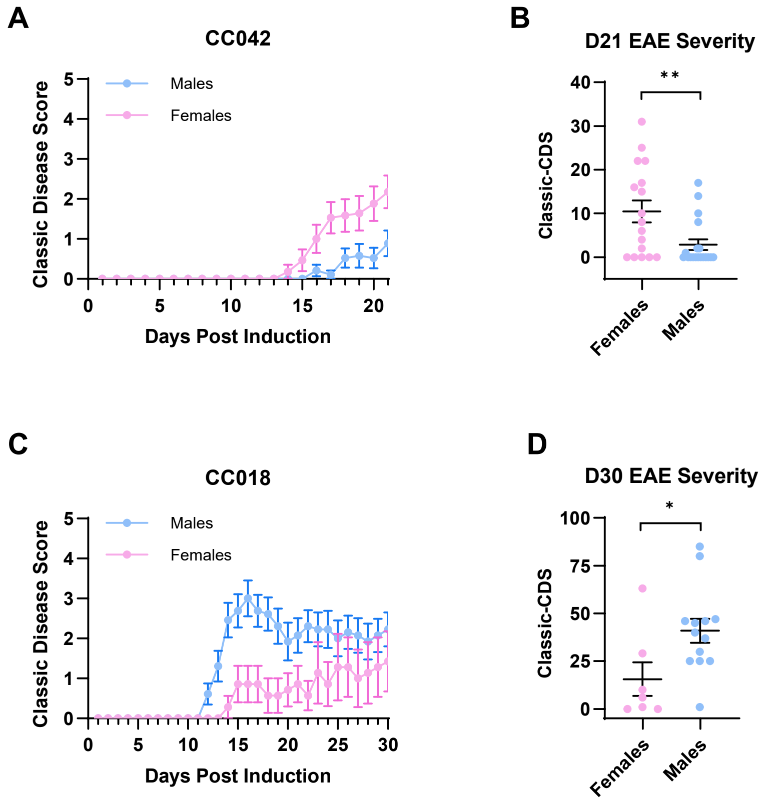

Supplemental Figure 9. Replicate experiments support sex effects in EAE severity in CC042 and CC018. EAE was induced and evaluated in additional cohorts of CC042 (12F + 14M) and CC018 (3F + 8M) mice, as described in Methods. Disease course data and CDS for each strain was pooled with the corresponding strain data from the original screen (Supplemental Table 2 and Supplemental Figure 5) for all the days for which data were available in both cohorts. (**A**) Compiled classic-EAE 21-day disease course and (**B**) distribution of classic-CDS between sexes for CC042 (total of 17F + 19M). (**C**) Compiled classic-EAE 30-day disease course and (**D**) distribution of classic-CDS between sexes for CC018 (total of 7F + 13M). Significance of differences of classic-CDS between sexes was determined by unpaired t test and indicated by asterisks where significant.

Supplemental Figure 10

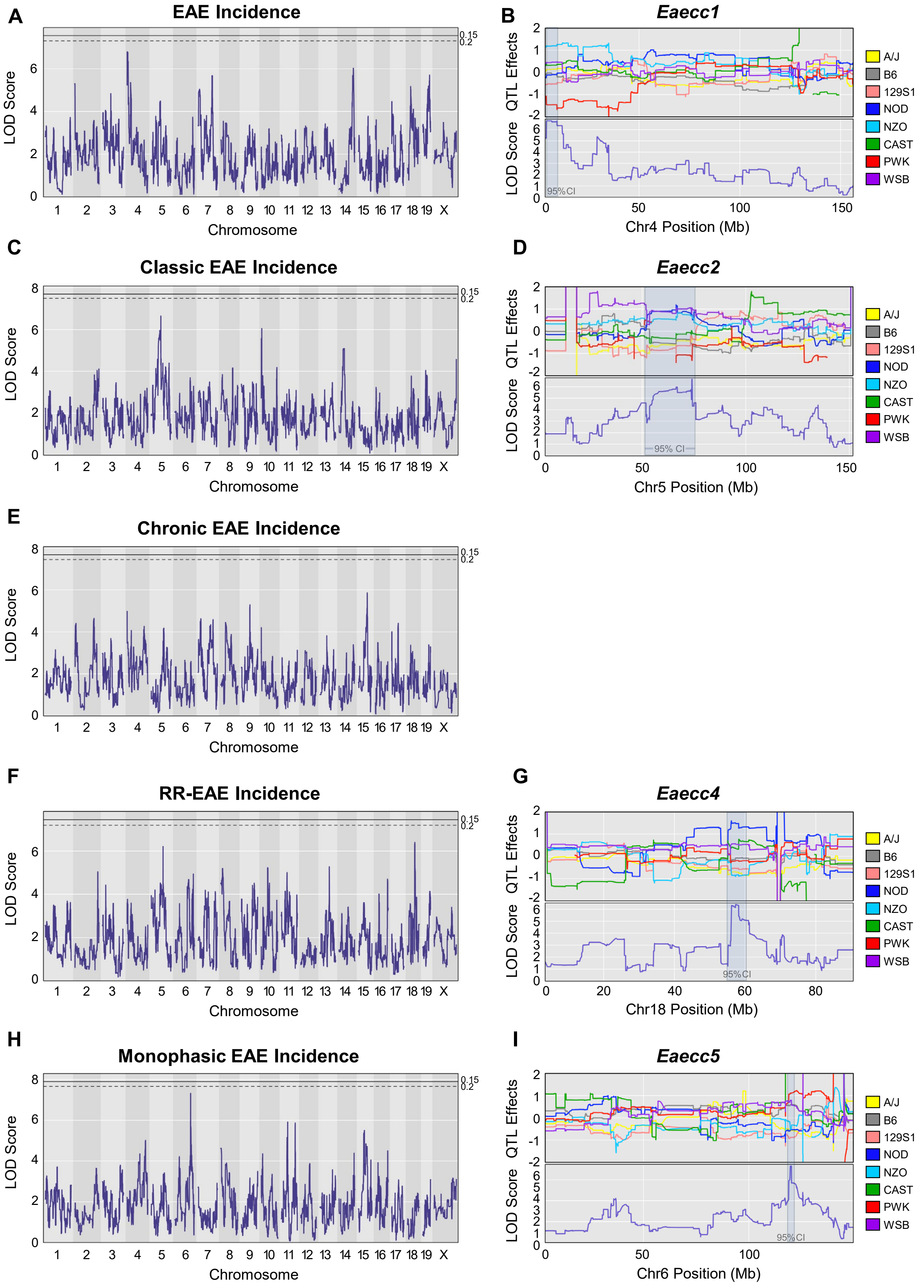

Supplemental Figure 10. QTL analysis reveals distinct genetic linkage patterns for several EAE incidence traits. EAE was induced and evaluated, as described in Figure 1. EAE disease phenotypes and QTVs were calculated, and QTL mapping was performed as described in methods. (**A**) Manhattan plot demonstrating LOD traces for total EAE incidence, and (**B**) corresponding CC founder allele effects plot for lead QTL identified on Chr4 -*Eaecc1*. (**C**) Manhattan plot demonstrating LOD traces for classic-EAE incidence, and (**D**) corresponding CC founder allele effects plot for lead QTL identified on Chr5 – *Eaecc2*. (**E**) Manhattan plot demonstrating LOD traces for chronic-EAE incidence. (**F**) Manhattan plot demonstrating LOD traces for RR-EAE incidence, and (**G**) corresponding CC founder allele effects plot for lead QTL identified on Chr18 – *Eaecc4*. (**H**) Manhattan plot demonstrating LOD traces for monophasic-EAE incidence, and (**I**) corresponding CC founder allele effects plot for lead QTL identified on Chr6 – *Eaecc5*. For Panels (**A**), (**C**), (**E**), (**F**) and (**H**), genome wide significance thresholds of 15% (solid line) and 20% (dashed line) were determined by permutations (n=1000).

Supplemental Figure 11

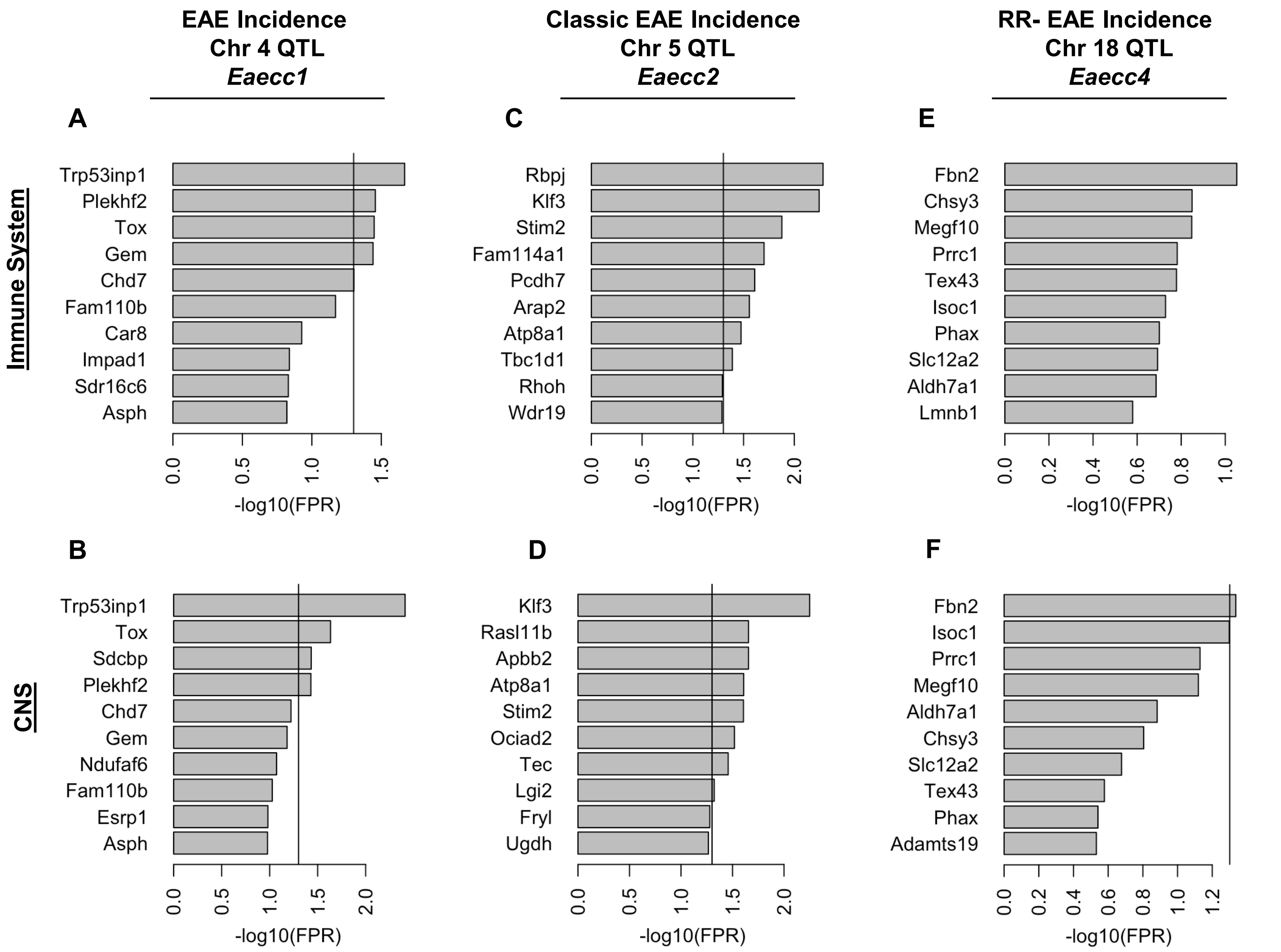

Supplemental Figure 11. Functional candidate gene prioritization nominates distinct genes associated with unique EAE incidence traits. SVM classifiers ranked gene candidates associated with EAE incidence QTLs in the context of either the CNS or immune system as described in Figure 8 and methods. Ranked candidate genes for: *Eaecc1* (EAE incidence) in the (**A**) immune system and (**B**) CNS, *Eaecc2* (classic-EAE incidence) in the (**C**) immune system and (**D**) CNS, and *Eaecc4* (RR-EAE incidence) in the (**E**) immune system and (**F**) CNS. The solid line in panels (**A**–**F**) corresponds to the FPR threshold of 0.05.

Supplemental Table 1. CC Founder Strain H2 Haplotypes

CC Founder Strain	H2 Haplotype
129S1/SvImJ	b
A/J	a
C57BL/6J	b
NOD/ShiLtJ	g7
NZO/HlLtJ	z
CAST/EiJ	novel
PWK/PhJ	novel
WSB/EiJ	novel

Supplemental Table 2. EAE QTVs by strain and sex

CC Strain	CDS[A]			Classic-CDS[A]			AR-CDS[A]			EAE Incidence[B]			Classic-EAE Incidence[B]			AR-EAE Incidence[B]			Chronic-EAE Incidence[B]			RR-EAE Incidence[B]			Monophasic-EAE Incidence[B]		
	Strain	Males	Females	Strain	Males	Females	Strain	Males	Females	Strain	Males	Females	Strain	Males	Females	Strain	Males	Females	Strain	Males	Females	Strain	Males	Females	Strain	Males	Females
CC001/Unc	66	49	79	63	49	75	6	0	10	78	75	80	78	75	80	0	0	0	44	50	40	22	0	40	11	25	0
CC002/Unc	45	56	30	42	53	29	4	7	1	91	83	100	82	83	80	9	0	20	18	33	0	64	50	80	9	0	20
CC003/Unc	33	42	24	26	28	24	8	16	0	80	100	60	70	80	60	10	20	0	10	20	0	30	40	20	40	40	40
CC004/TauUnc	139	142	137	29	32	25	113	111	114	93	86	100	14	14	14	79	71	86	64	71	57	14	14	14	14	0	29
CC006/TauUnc	21	26	16	18	26	10	3	0	6	80	100	60	70	100	40	10	0	20	10	0	20	50	80	20	20	20	20
CC010/GeniUnc	45	37	54	45	37	54	0	0	0	80	80	80	80	80	80	0	0	0	40	40	40	40	40	40	0	0	0
CC011/Unc	1	0	1	1	0	1	0	0	0	10	0	20	10	0	20	0	0	0	0	0	0	0	0	0	10	0	20
CC018/Unc	34	53	11	34	53	11	0	0	0	67	80	50	67	80	50	0	0	0	22	40	0	22	20	25	22	20	25
CC020/GeniUncJ	2	3	2	1	2	0	1	1	2	40	60	20	20	40	0	20	20	20	0	0	0	0	0	0	40	60	20
CC023/GeniUnc	22	17	27	22	17	27	0	0	0	70	80	60	70	80	60	0	0	0	40	40	40	20	20	20	10	20	0
CC028/GeniUnc	151	183	119	140	183	97	13	0	26	90	100	80	80	100	60	10	0	20	80	80	80	10	20	0	0	0	0
CC030/GeniUnc	93	90	96	92	88	96	6	9	2	100	100	100	100	100	100	0	0	0	100	100	100	0	0	0	0	0	0
CC031/GeniUnc	26	12	38	26	11	38	0	1	0	78	50	100	78	50	100	0	0	0	22	0	40	11	25	0	44	25	60
CC032/GeniUnc	27	35	19	25	33	17	2	2	2	90	80	100	70	60	80	20	20	20	20	40	0	10	0	20	60	40	80
CC036/Unc	18	9	24	14	9	18	5	0	8	70	50	83	60	50	67	10	0	17	10	0	17	20	25	17	40	25	50
CC037/TauUnc	79	90	68	73	86	61	6	5	7	90	100	80	90	100	80	0	0	0	40	60	20	40	40	40	10	0	20
CC038/GeniUnc	25	50	0	7	14	0	23	46	0	30	60	0	0	0	0	30	60	0	20	40	0	10	20	0	0	0	0
CC040/TauUnc	12	20	6	12	20	6	5	12	0	33	25	40	33	25	40	0	0	0	33	25	40	0	0	0	0	0	0
CC041/TauUnc	95	101	90	95	99	90	1	2	0	100	100	100	90	80	100	10	20	0	100	100	100	0	0	0	0	0	0
CC042/GeniUnc	71	37	104	70	37	104	1	0	1	70	60	80	70	60	80	0	0	0	60	40	80	10	20	0	0	0	0
CC043/GeniUnc	104	98	110	104	98	111	2	0	3	100	100	100	100	100	100	0	0	0	60	40	80	40	60	20	0	0	0
CC044/Unc	4	1	6	4	6	1	0	0	0	50	80	20	50	80	20	0	0	0	0	0	0	0	0	0	50	80	20
CC046/Unc	57	105	10	57	105	9	0	0	0	90	100	80	90	100	80	0	0	0	30	60	0	40	40	40	20	0	40
CC051/TauUnc	32	52	12	32	51	12	1	2	0	80	100	60	80	100	60	0	0	0	0	0	0	60	80	40	20	20	20
CC059/TauUnc	41	36	46	40	35	45	1	0	1	90	80	100	90	80	100	0	0	0	20	40	0	60	20	100	10	20	0
CC061/GeniUnc	44	45	43	33	34	33	21	31	10	100	100	100	73	67	80	27	33	20	18	33	0	27	17	40	55	50	60
CC068/GeniUnc	6	6	7	6	6	7	0	0	0	78	100	60	78	100	60	0	0	0	0	0	0	11	0	20	67	100	40
CC072/GeniUnc	20	0	39	0	0	0	20	0	39	30	0	60	0	0	0	30	0	60	20	0	40	10	0	20	0	0	0
CC074/Unc	23	29	18	23	28	18	1	1	0	80	100	60	80	100	60	0	0	0	20	20	20	30	60	0	30	20	40
CC075/Unc	62	53	70	55	50	61	18	17	19	70	80	60	60	80	40	10	0	20	40	20	60	20	40	0	10	20	0
CC083/Unc	71	60	82	39	26	51	32	34	31	100	100	100	60	60	60	40	40	40	70	80	60	20	20	20	10	0	20
CC084/TauUnc	79	81	77	63	73	53	44	26	62	100	100	100	70	80	60	30	20	40	50	40	60	40	60	20	10	0	20

[A]CDS values are presented as averages (for strain, males, and females) and are rounded to nearest whole number

[B]Incidence values reported as percent and rounded to nearest whole number

Supplemental Table 3. Sex differences in EAE disease severity

CC Strain	CDS			Classic-CDS			AR-CDS		
	Sex Difference	P-adj[A]	P-raw[B]	Sex Difference	P-adj[A]	P-raw[B]	Sex Difference	P-adj[A]	P-raw[B]
CC001/Unc	None	>0.9999	0.2691	None	>0.9999	0.3216	None	>0.9999	0.5899
CC002/Unc	None	>0.9999	0.283	None	>0.9999	0.3041	None	>0.9999	0.7416
CC003/Unc	None	>0.9999	0.4801	None	>0.9999	0.8778	None	>0.9999	0.3883
CC004/TauUnc	None	>0.9999	0.8267	None	>0.9999	0.7375	None	>0.9999	0.8795
CC006/TauUnc	None	>0.9999	0.7005	None	>0.9999	0.5174	None	>0.9999	0.7282
CC010/GeniUnc	None	>0.9999	0.4899	None	>0.9999	0.4765	None	>0.9999	>0.9999
CC011/Unc	None	>0.9999	0.9562	None	>0.9999	0.9548	None	>0.9999	>0.9999
CC018/Unc	None	0.9836	0.1205	None	0.9755	0.1095	None	>0.9999	0.6648
CC020/GeniUncJ	None	>0.9999	0.9687	None	>0.9999	0.9419	None	>0.9999	0.9642
CC023/GeniUnc	None	>0.9999	0.6947	None	>0.9999	0.6857	None	>0.9999	>0.9999
CC028/GeniUnc	M > F	0.3261	0.0123	M > F	0.0189	0.0006	None	0.9943	0.1489
CC030/GeniUnc	None	>0.9999	0.8138	None	>0.9999	0.7708	None	>0.9999	0.6948
CC031/GeniUnc	None	>0.9999	0.3345	None	>0.9999	0.3106	None	>0.9999	0.9789
CC032/GeniUnc	None	>0.9999	0.5353	None	>0.9999	0.5227	None	>0.9999	>0.9999
CC036/Unc	None	>0.9999	0.5728	None	>0.9999	0.7212	None	>0.9999	0.6604
CC037/TauUnc	None	>0.9999	0.3818	None	>0.9999	0.3238	None	>0.9999	0.9374
CC038/GeniUnc	None	0.8242	0.0529	None	>0.9999	0.5821	M > F	0.2694	0.0098
CC040/TauUnc	None	>0.9999	0.6006	None	>0.9999	0.5893	None	>0.9999	0.5175
CC041/TauUnc	None	>0.9999	0.6546	None	>0.9999	0.6917	None	>0.9999	0.893
CC042/GeniUnc	M < F	0.2437	0.0087	M < F	0.1951	0.0068	None	>0.9999	0.9732
CC043/GeniUnc	None	>0.9999	0.6238	None	>0.9999	0.6045	None	>0.9999	0.8665
CC044/Unc	None	>0.9999	0.8199	None	>0.9999	0.8144	None	>0.9999	>0.9999
CC046/Unc	M > F	0.0074	0.0002	M > F	0.0045	0.0001	None	>0.9999	0.9821
CC051/TauUnc	None	0.9844	0.1219	None	0.9839	0.121	None	>0.9999	0.9285
CC059/TauUnc	None	>0.9999	0.6832	None	>0.9999	0.7037	None	>0.9999	0.9553
CC061/GeniUnc	None	>0.9999	0.9347	None	>0.9999	0.9843	None	0.9995	0.2116
CC068/GeniUnc	None	>0.9999	0.9897	None	>0.9999	0.9893	None	>0.9999	>0.9999
CC072/GeniUnc	None	0.9859	0.1248	None	>0.9999	>0.9999	M < F	0.6066	0.0287
CC074/Unc	None	>0.9999	0.6717	None	>0.9999	0.6977	None	>0.9999	0.9464
CC075/Unc	None	>0.9999	0.5174	None	>0.9999	0.6562	None	>0.9999	0.9019
CC083/Unc	None	>0.9999	0.3796	None	>0.9999	0.3044	None	>0.9999	0.8665
CC084/TauUnc	None	>0.9999	0.8783	None	>0.9999	0.4139	M < F	0.7382	0.041

[A]P-adj: Reported p value as determined by two-way ANOVA using Šídák's multiple comparisons

[B]P-raw: Reported p value as determined by two-way ANOVA using Fishers LSD multiple comparisons

Supplemental Table 4. Antibodies used for flow cytometry

Antigen	Fluorophore	Experiment/Figure	Supplier	Clone	Catalog #
Live/Dead	UV-Blue	Figure 5 + 6	Invitrogen	-	L34962A
CD45	AF700	Figure 5	BioLegend	30-F11	103128
CD11b	APC-Fire 750	Figure 5 + 6	BioLegend	M1/70	101262
TCRβ	BV605	Figure 5 + 6	BioLegend	H57-597	109241
Ly6G	BV510	Figure 5 + 6	BioLegend	1A8	127633
CD19	PE-Cy5	Figure 5	BioLegend	6D5	115510
CX3CR1	PE-Dazzle 594	Figure 5 + 6	BioLegend	SA011F11	149014
CD8	PE-Cy7	Figure 5 + 6	BioLegend	53-6.7	100722
CD4	A488	Figure 5	BioLegend	GK1.5	100423
GM-CSF	PE	Figure 5	BioLegend	MP1-22E9	505406
IFNγ	AF647	Figure 5	BioLegend	XMG1.2	505814
IL-17	BV421	Figure 5	BioLegend	TC11-18H10.1	506926
CD45.1	APC	Figure 6	BioLegend	A20	110714
CD45.2	PerCP-Cy5.5	Figure 6	BioLegend	104	109828
CD19	PE	Figure 6	BioLegend	1D3/CD19	152408
CD4	BV421	Figure 6	BioLegend	GK1.5	100443
